# Smart antenna with reconfigurable polarization for future generation of mm-wave communication

**DOI:** 10.1038/s41598-025-22771-z

**Published:** 2025-11-04

**Authors:** Mahmoud A. Mohamed, Abdelhamied A. Ateya, Khalid F. A. Hussein, Asmaa E. Farahat, Walid S. El-Deeb

**Affiliations:** 1 Electronics and Communications Engineering Department, Faculty of Engineering, Zagazig University, Zagazig, 44519 Egypt; 2 Microwave Engineering Department, Electronics Research Institute (ERI), Cairo, 11843 Egypt

**Keywords:** Smart antenna, Reconfigurable antenna, Circular polarization, Millimeter-wave communication, PIN diode switching, Dual-band operation, Engineering, Physics

## Abstract

Reconfigurable antennas with polarization agility are critical for modern millimeter-wave (mm-wave) communication systems, enabling improved link reliability, interference mitigation, and system adaptability. This paper presents a dual-band, polarization-reconfigurable microstrip antenna based on a square patch notched at two opposite corners and loaded with four PIN diodes. A defected ground structure (DGS) is introduced to enhance circular polarization purity and extend bandwidth. The antenna operates over two frequency bands: 27–29 GHz (lower band) and 32–32.6 GHz (upper band). By appropriately biasing the PIN diodes, the antenna can generate either left-hand circular polarization (LHCP) or right-hand circular polarization (RHCP) in the lower band, while maintaining linear polarization in the upper band, providing multifunctional operation within a compact design. Simulated results confirm impedance matching in the 27–29 GHz band, with a 3 dB axial ratio bandwidth of 27.42–28.22 GHz and a minimum axial ratio of 0.2 dB near 28 GHz, indicating excellent polarization purity. The antenna achieves peak gain above 6.2 dBic in both circular polarizations at the lower band and 7.2 dBi in the upper band with linear polarization. Efficiencies reach 84% at 28 GHz and over 76% at 32.3 GHz. The antenna, feed network, defected ground structure, and biasing circuits are integrated in a three-layer PCB with sub-50 μm precision traces enabled by LPKF laser prototyping, ensuring minimal parasitics. Experimental measurements show good agreement with simulations. These results validate the proposed antenna as an efficient, compact, and versatile solution for polarization-reconfigurable mm-wave communication, with advantages in adaptability, integration, and performance stability.

## Introduction

Smart antennas are emerging as critical components in future-generation millimeter-wave communication systems, offering adaptive polarization control, interference mitigation, and improved link resilience^1–3^. Reconfigurability, adaptivity, and smartness are interrelated concepts that collectively define the intelligence of next-generation antenna systems. Reconfigurability refers to the antenna’s ability to alter its operating parameters, such as frequency, polarization, or radiation pattern, through integrated mechanisms like switches or tunable materials. Adaptivity builds on this by enabling real-time changes in response to varying environmental or system conditions, often guided by control algorithms or feedback loops. Smart antennas leverage both traits to autonomously optimize communication performance, enhance spectrum utilization, and mitigate interference, making them indispensable for future wireless systems^4,5^.

Unlike traditional array-based smart antennas that depend heavily on signal processing techniques^6,7^, reconfigurable single-element antennas achieve embedded intelligence by dynamically altering their electromagnetic response through integrated switching mechanisms^8–10^. This inherent adaptability is particularly advantageous in cluttered or mobile environments, where issues such as polarization mismatch and multipath fading can significantly degrade link quality. Additionally, reconfigurable single-element antennas facilitate the design of compact multiple-input multiple-output (MIMO) systems, enhancing communication robustness even in the presence of channel impairments.

However, implementing polarization reconfigurability at mm-wave frequencies presents significant challenges. The integration of active components such as PIN diodes introduces parasitic capacitance and resistance, which can degrade polarization purity and impedance matching^10^. Such antennas require precise fabrication of fine geometries to support sub-100 μm feed structures, which is essential to maintain performance at high frequencies^11,12^. Moreover, achieving dual-band operation with both circular polarization (CP) and linear polarization (LP) typically requires complex feeding networks or multiple feed ports, leading to increased size, cost, and control complexity^13,14^.

Several prior works have explored polarization reconfigurable antennas, For example the work of^15^ proposes a designed of multi-polarization square patch at 2.45 GHz, using Wilkinson dividers and PIN diodes to switch between LHCP, RHCP, and linear polarization modes. In^16^, the authors demonstrate PIN-diode-based metasurface antennas for beam switching at mm-wave frequencies, though their focus was on pattern switching rather than polarization. The authors of^17^ developed a dual-band reconfigurable antenna switching between sub-6 GHz and mm-wave using a single PIN diode; however, their design did not support polarization diversity.

Recent advances in reconfigurable antennas have focused on achieving compact designs, enhanced frequency agility, and practical integration for modern wireless systems. For example, a low-loss paper-substrate triple-band frequency reconfigurable microstrip antenna has been reported for sub-7 GHz applications, demonstrating efficient performance with environmentally friendly materials^18^. Similarly, electronically reconfigurable and conformal triband antennas have been introduced for portable and flexible communication devices^19^. Moreover, wide, dual-, and single-band frequency reconfigurable antennas with compact layouts have been realized to meet the requirements of next-generation wireless systems^20^. These recent works highlight the diverse approaches to frequency reconfigurability and motivate the design presented in this study.

Moreover, recent studies have reported polarization-reconfigurable metasurface antennas that integrate diode or resistor control to achieve wideband operation while simultaneously reducing radar cross-section (RCS). In^21^, Ding et al. demonstrate a wideband dual-polarized metasurface antenna whose polarization states are reconfigured using PIN diodes and resistors, while maintaining low RCS characteristics. Similarly, in^22^, the same group presents a low-profile wideband metasurface antenna with resistor-based polarization reconfiguration and significantly reduced RCS, optimized for wireless communication applications. These works highlight the effectiveness of biasing and control techniques in enabling multifunctional reconfigurable antennas, providing context and motivation for the design approach adopted in this paper.

In contrast to previous designs, the proposed antenna delivers hybrid reconfigurability, supporting LHCP or RHCP in the lower band and linear polarization in the upper band, using a single feed line and only four PIN diodes. This approach minimizes footprint, complexity, and RF losses, while offering dual-band and dual-polarization flexibility without external $\:90^\circ\:$ hybrids or multiple feed ports. The antenna is based on a square microstrip patch notched at two opposite corners, which allows CP generation via orthogonal mode perturbation. Four PIN diodes are strategically placed near the notches and feed branches to control the excitation edge and enable polarization switching. The feed network is carefully designed using sub-$\:100\:{\upmu\:}\text{m}$ precision traces enabled by LPKF laser prototyping, ensuring accurate impedance matching and minimal parasitics. The result is a mm-wave antenna capable of producing: (i) impedance matching over the frequency bands $\:27-29\:\text{G}\text{H}$z and $\:32-32.6\:\text{G}\text{H}\text{z}$, (ii) circular polarization with reconfigurable sense (LHCP or RHCP) over the frequency $\:27.42\--28.22\:\text{G}\text{H}\text{z}$ (iii) minimum axial ratio of $\:\sim0.2\:\text{d}\text{B}$ at $\:28\:\text{G}\text{H}\text{z}$, and gain exceeding $\:6\:\text{d}\text{B}\text{i}\text{c}$, (iv) linear polarization over $\:32\--32.6\:\text{G}\text{H}\text{z}$, with gain exceeding $\:7\:\text{d}\text{B}\text{i}$, and (v) high radiation efficiency ($\:84\text{\%}\:\text{a}\text{t}\:28\:\text{G}\text{H}\text{z}$ and $\:82\%$ at $\:32.3\:\text{G}\text{H}\text{z}$).

In summary, the contributions of this work include: (i) a dual-band polarization-reconfigurable antenna achieving CP in the lower band and LP in the upper band using a compact, single-port feed network, (ii) novel integration of only four PIN diodes to control polarization sense without external hybrids, (iii) Implementation on Rogers RO3003 with fine feature geometry achieved by LPKF laser fabrication, delivering high performance at mm-wave frequencies, and (iv) comprehensive simulation and experimental validation confirming impedance, AR bandwidths, gain, and efficiency.

The remainder of the paper is structured as follows: Sect. 2 details the antenna geometry and switching mechanism; Sect. 3 describes fabrication and measurement procedures; Sect. 4 presents performance results; and Sect. 5 provides a comparative analysis and Sect. 6 summarizes the concluding remarks.

## Antenna and reconfigurable feeding network design

The proposed antenna is based on a square microstrip patch designed to operate in the millimeter-wave frequency band to provide circular polarization with electronically controllable sense at 28 GHz and linear polarization at $\:31.5\:\text{G}\text{H}\text{z}$.

### Patch design and impedance matching

**Fig. 1:**
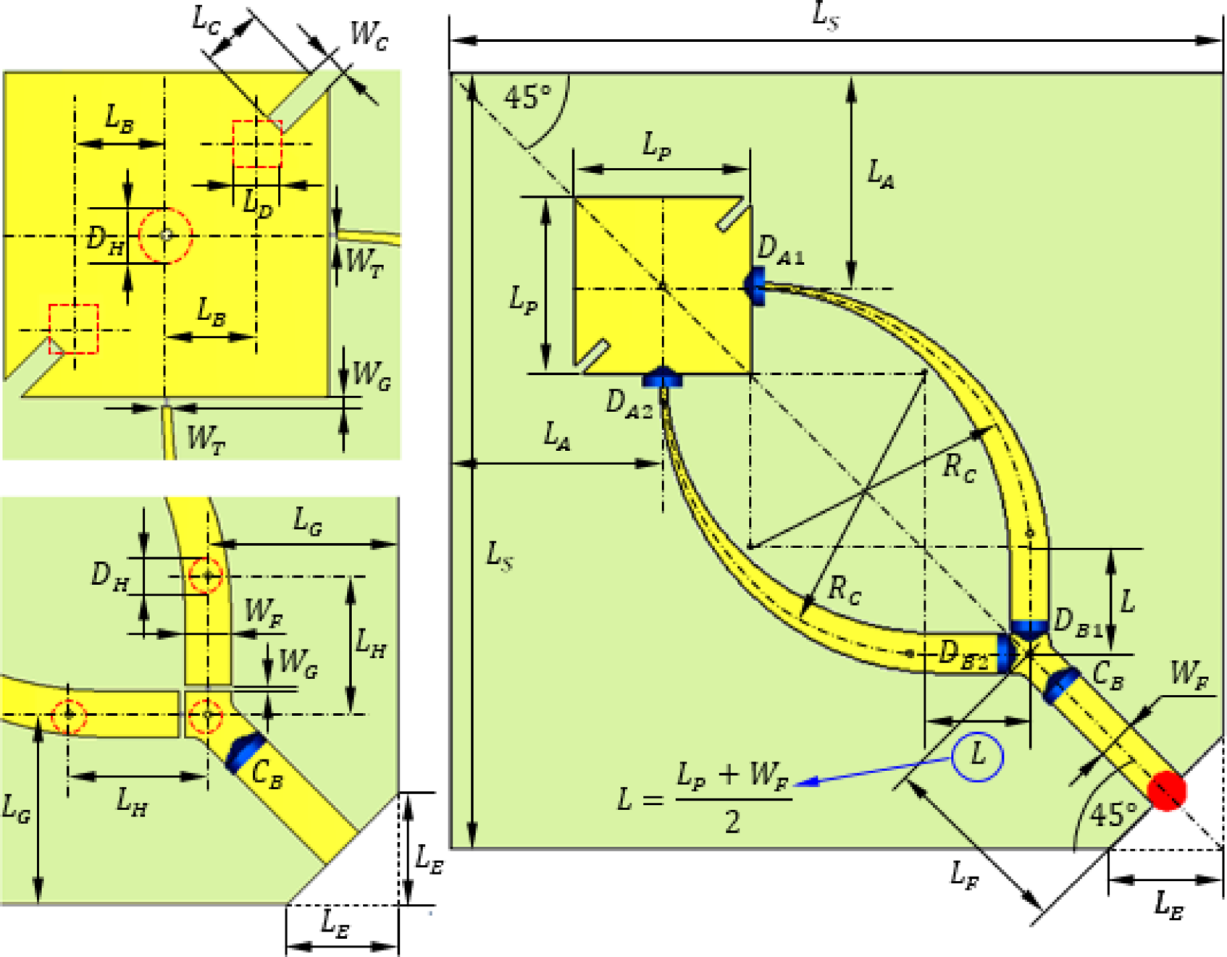
Design of the proposed antenna and reconfigurable feed network with zoomed views at the patch and the branch point of the feed network. The PIN diodes ($\:{D}_{A1}$, $\:{D}_{B1}$ $\:{D}_{A2}$, and $\:{D}_{B2}$) are indicated in blue color. The holes cut in the ground plane are drawn using dashed red lines.

As shown in Fig. 1, circular polarization is achieved by etching two rectangular notches at diagonally opposite corners of the patch. Additionally, two square cuts are etched in the ground structure for further improvement of circular polarization. These perturbations of both the patch and the ground facilitate the excitation of orthogonal modes $\:{\text{T}\text{M}}_{10}$​ and $\:{\text{T}\text{M}}_{01}$with a 90° phase difference, resulting in CP radiation.

To get a rectangular patch resonant at a specific frequency, the effective length is given by the following expression^23^,

1 $$\:{L}_{eff}=\frac{c}{2{f}_{r}\sqrt{{\epsilon}_{eff}}}$$

where

2 $$\:{\epsilon}_{eff}=\frac{{\epsilon}_{r}+1}{2}+\frac{{\epsilon}_{r}-1}{2}{\left(1+12\frac{h}{W}\right)}^{-\frac{1}{2}}$$

where $\:{\epsilon}_{r}$​ is the substrate permittivity, $\:h$ is substrate thickness, and $\:W$ is patch width (which is the same as the patch length).

To achieve efficient power transfer and minimize reflection at the antenna input, impedance matching is performed by inserting a tapered microstrip section between the main uniform feed line and the radiating patch. This tapered section acts as a gradual impedance transformer, enabling a smooth transition between the characteristic impedance of the feed line and the input impedance of the patch.

Importantly, this tapered feed is adopted instead of a conventional inset feed, which typically requires shifting the feed point toward the center of the patch. Such offset would break the geometrical symmetry of the radiating structure about its 45° diagonal axis. Maintaining this symmetry is critical for the balanced excitation of the orthogonal $\:{\text{T}\text{M}}_{10}$​ and $\:{\text{T}\text{M}}_{01}$​ modes, and thus for achieving high-purity circular polarization.

The tapering rate is defined by a linear variation of the microstrip line width from $\:{W}_{F}$​, the width corresponding to a $\:50\:{\Omega\:}$ characteristic impedance, to a narrower width $\:{W}_{T}$​ near the patch edge. This width transition occurs over a taper length $\:{L}_{T}$​, which is selected based on transmission line matching theory to optimize the impedance transformation.

Geometrically, the tapered region is shaped as a quarter-circular arc, chosen for compactness and effective spatial routing. The radius $\:{R}_{C}$​ of the arc is carefully determined such that the length of the curved centerline of the arc equals $\:{L}_{T}$​, thereby ensuring the desired physical length for impedance transformation is preserved along the arc. This configuration guarantees that the electrical performance of the taper matches that of an equivalent straight tapered line while conforming to the layout constraints of the antenna.

This approach not only achieves high return loss performance but also contributes to the preservation of polarization symmetry and manufacturability, making it well-suited for high-frequency circularly polarized antenna implementations.

### Feed network design

The geometry of the proposed antenna and the reconfigurable feeding network are shown in Fig. 1. To enable dynamic polarization reconfigurability between left-hand circular polarization (LHCP) and right-hand circular polarization (RHCP), a dual-branch microstrip feeding network is employed. The main microstrip line is split into two branches, each capable of exciting the patch with a distinct phase configuration corresponding to LHCP or RHCP. The switching between branches is controlled by four PIN diodes integrated into the feed structure.

Each branch of the feed line is composed of two regions, a tapered curved region and a uniform straight region. A gap is cut between one side of the square patch and the neck (narrowest end) of the tapered feed line region. This gap can be bridged by activating a PIN diode ($\:{D}_{1A})$. Another gap is cut between the other end (of the uniform straight region) and one end of the common feed line. This gap can be bridged by activating a PIN diode ($\:{D}_{1B}$).

Branch 1 of the feeding line has two gaps “A1” and “B1” that can be bridged by activating $\:{D}_{A1}$ and $\:{D}_{B1}$, respectively. Branch 2 of the feeding line has two gaps “A2” and “B2” that can be bridged by activating $\:{D}_{A2}$ and $\:{D}_{B2}$, respectively.

Thus, each branch is connected to the main feeder through one PIN diode ($\:{D}_{B1}$) for branch 1, and $\:{D}_{B2}$ for Branch 2. Forward-biasing a diode creates a low-impedance path that activates the corresponding branch, while the other set remains in the reverse-biased (high-impedance) state to isolate the inactive path. Additionally, a single PIN diode ($\:{D}_{A1}$ or $\:{D}_{A2}$) is placed near the connection point of each branch to the patch periphery. These diodes determine which edge of the patch is excited, thereby controlling the phase progression necessary to establish the desired polarization sense.

For LHCP operation, diodes $\:{D}_{A1}$ and $\:{D}_{B1}$ are forward-biased (“ON”), while $\:{D}_{A2}$ and $\:{D}_{B2}$ are in the “OFF” state. Conversely, for RHCP, the roles are reversed, diodes $\:{D}_{A2}$ and $\:{D}_{B2}$ are activated, and the diodes $\:{D}_{A1}$ and $\:{D}_{B1}$ are deactivated. This electronically controlled switching mechanism enables fast and robust polarization reconfiguration without the need for mechanical rotation or complex analog phase-shifting networks.

In the simulations, the PIN diodes are represented as dual-state (ON/OFF) switches characterized by their corresponding impedance values^24^.

The impedance of a forward-biased PIN diode (ON state) can be calculated as,

3 $$\:{Z}_{ON}\approx\:{R}_{S}+j{L}_{P}$$

where $\:{R}_{S}$​ is the forward-bias (“series”) resistance, decreasing with increasing forward bias current. The parasitic inductance ($\:{L}_{P}$) is usually small, sometimes neglected depending on frequency.

On the other hand, the impedance of a reverse biased PIN diode (OFF state) can be calculated as,

4 $$\:{Z}_{OFF}=\frac{1}{j\omega\:{C}_{j}}\parallel\:{R}_{P}$$

where $\:{C}_{j}$ is the junction (or depletion-region) capacitance, and $\:{R}_{P}$​ is a (large) parallel resistance modeling leakage or loss. In many designs the capacitive reactance dominates in OFF state at high frequencies.

Each feed branch consists of two segments: a tapered curved section for impedance matching and a uniform straight section for stable propagation. Two gaps are introduced along each branch: (i) Gap “A1” and gap “B1” for Branch 1, bridged by diodes $\:{D}_{A1}$ and $\:{D}_{B1}$​, respectively. (ii) Gap “A2” and gap “B2” for Branch 2, bridged by diodes $\:{D}_{A2}$ and $\:{D}_{B2}$​, respectively. Diodes $\:{D}_{B1}$ and $\:{D}_{B2}$​ control the connection between the respective branches and the main feed line, while $\:{D}_{A1}$ and $\:{D}_{A2}$ govern the connection to the patch periphery. By selectively biasing these diodes, the desired polarization mode is achieved.

For LHCP operation, diodes ​ and $\:{D}_{A1}$ and $\:{D}_{B1}$​​ are forward-biased (“ON”), enabling Branch 1, while $\:{D}_{A2}$ and $\:{D}_{B2}$ remain reverse-biased (“OFF”). For RHCP, $\:{D}_{A2}$ and $\:{D}_{B2}$ are activated, and $\:{D}_{A1}$ and $\:{D}_{B1}$​​ are deactivated. This selective activation provides a low-impedance path along the chosen branch, effectively exciting the patch edge with the appropriate phase configuration.

This design enables rapid and reliable electronic switching between LHCP and RHCP modes without the need for mechanical components or complex phase-shifting networks, making it highly suitable for compact and high-speed millimeter-wave communication systems.

The dimensions of the proposed antenna integrated with the feed network and the biasing circuit are listed in (Table 1). It should be noted that these dimensions are optimized through extensive parametric study performed by simu8lation to achieve the antenna design goals. Some examples of such parametric study are presented in Sect. 2.5.

### Bias network design and layout

**Fig. 2:**
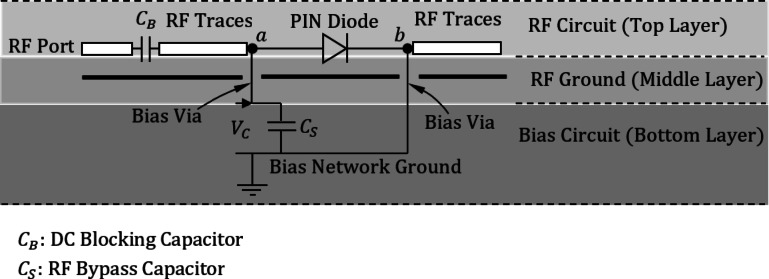
Schematic of the biasing circuit (on the bottom layer) showing the interconnections across the stacked three-layer structure.

To isolate the RF feed network from the DC bias circuitry, a three-layer PCB stack-up was adopted. The antenna and feed network are implemented on the top layer, while a continuous ground plane occupies the middle layer. The bias network, including series current-limiting resistors and bias traces, is routed on the bottom layer. The middle-layer ground serves as an effective RF shield, minimizing undesired coupling between the bias lines and the radiating elements. Vias connect the bottom-layer bias lines to the PIN diodes located on the top layer, ensuring that the diodes receive stable biasing currents without degrading the antenna’s RF performance. Electromagnetic co-simulations and measurements confirmed that the multilayer bias network introduces negligible degradation to impedance matching and radiation characteristics (see Fig. 2).

We intentionally use an isolated bias return on the bottom layer (i.e., the bias return is not electrically tied to the antenna RF ground/middle ground plane). This design permits the bias network to be referenced to a separate DC rail while the middle layer remains the RF ground for the antenna. A shunt capacitor ($\:{C}_{s}=1\:\text{p}\text{F}$) is connected between the biasing vias and the bias return for stabilization and ripple removing for the biasing voltages.

A discrete RF choke inductor is not strictly required because: (i) Ground-plane isolation:

The middle-layer continuous ground already shields the bias lines (bottom layer) from the RF network (top layer). The only RF-sensitive path is the short via that connects the diode to its bias pad. (ii) Bias feed as high impedance: Since the bias traces are routed on the bottom layer and connect through vias, they are inherently very short at the diode terminals. These traces are narrow and long enough to act as a high-impedance line at mm-wave frequencies essentially serving the same role as an RF choke.

The PIN diodes are biased from a regulated DC rail of $\:3\--5\:\text{V}$, with forward current limited by a series resistor $\:{R}_{B}$​ placed on the bias layer as close as possible to the via feeding the diode. Typical resistor values lie in the $\:68\--470\:{\Omega\:}$ range depending on the desired forward current ($\:10\--50\:\text{m}\text{A}$). For a $\:3\--5\:\text{V}$ bias rail and a typical PIN diode forward voltage of $\:\approx\:1\:\text{V}$, a practical choice is $\:{R}_{B}$ $\:=\:220\:{\Omega\:}$, providing $\:\approx\:10\--15\:\text{m}\text{A}$ of forward current. Resistors must be rated for the expected dissipation and should be located immediately adjacent to the diode via on the bias layer. Where precise RF performance is critical, constant-current biasing or insertion-loss characterization versus bias current may be used to select the minimum forward current required to achieve the target insertion loss.

DC blocking capacitors and RF decoupling capacitors (small shunt capacitors appropriate for mm-wave operation) are employed at the RF ports as described elsewhere in the manuscript. A discrete RF choke inductor is not required for this design for two reasons: (i) Ground-plane isolation: The middle-layer ground plane shields the bias lines from the RF feed network. The only RF-sensitive path is the short via connecting each diode to its bias pad. (ii)High-impedance bias feed: The bottom-layer bias traces are narrow and short, effectively behaving as high-impedance lines at mm-wave frequencies and fulfilling the role of an RF choke.

The bias return on the bottom layer is intentionally isolated from the antenna RF ground (middle ground plane), allowing the bias network to be referenced to a separate DC supply while maintaining the middle layer as the RF ground. To stabilize the bias voltages and suppress ripple, a shunt capacitor $\:{C}_{s}=1\:$pF is connected between each diode bias via and the isolated bias return. This capacitor presents a low RF impedance path at mm-wave frequencies, ensuring that RF leakage into the bias network is minimized while maintaining full DC isolation between the antenna ground and the bias return.

A series DC-blocking capacitor $\:{C}_{B}$​ of $\:1.0\:\text{p}\text{F}$ (C0G/NP0, 0201/0402) is used at the RF port; at 28 GHz this yields a reactance of ≈ 5.7 Ω and an insertion loss ≲ 0.25 dB while keeping the high-pass cutoff well below the operating band. Values were optimized in EM co-simulation and confirmed by S-parameter measurements.

### Multilayer structure and integration of feeding, ground, and biasing networks

The proposed reconfigurable circularly polarized antenna is implemented using a three-layer stacked configuration, as illustrated in Figs. 1 and 2. This multilayer design enables the effective separation and integration of the radiating element, feeding network, biasing circuitry, and ground plane, ensuring compactness and high performance at millimeter-wave frequencies.

**Fig. 3:**
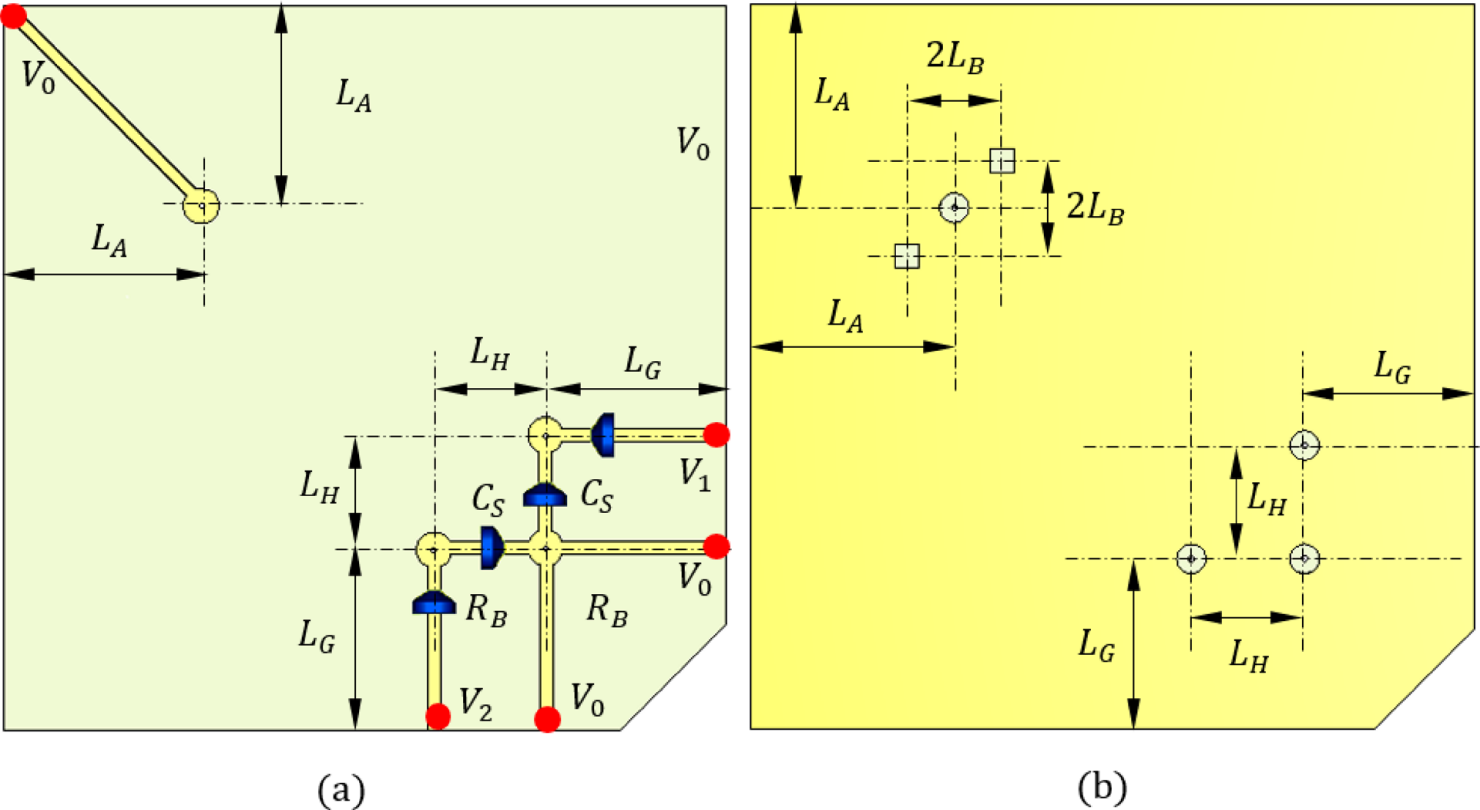
Design of (**a**) the biasing network on the bottom layer (3rd layer) and (**b**) the defected ground structure on the intermediate layer (2nd layer).

The top layer (first layer) hosts the square patch radiator along with the dual-branch feeding network responsible for polarization control. This layer includes the microstrip lines, rectangular notches, and the PIN diode control points required for LHCP/RHCP reconfiguration. The bottom layer (third layer) accommodates the biasing network, implemented as a planar DC circuit. As shown in Fig. 3(a), biasing voltages are applied at specific locations indicated by red dots. These points correspond to the diode control terminals and are used to switch the feed branches via applied DC voltages. The intermediate layer (second layer) serves as the defected ground structure (DGS), depicted in Fig. 3(b). Two square apertures of side length $\:{L}_{D}$​ are etched into the ground plane directly beneath the square patch. These apertures improve the axial ratio and enhance circular polarization quality by disturbing the image currents under the radiating patch.

To interface the biasing network with the diodes embedded in the top-layer feed structure, four vertical through-hole vias are employed. These vias pass through all three layers, connecting the DC bias lines on the bottom layer to the diode terminals on the top layer. In the ground layer, each via passes through a circular hole (shown as red dashed circles) with a diameter $\:{D}_{H}$​, which is intentionally made larger than the via diameter to avoid unintended electrical contact with the ground. This clearance ensures that the bias signals are isolated from the ground plane while preserving mechanical stability and manufacturing tolerance. The biasing voltages $\:{V}_{0}$ and $\:{V}_{1}$, and $\:{V}_{2}$ can be applied at the points shown in Fig. 3(a). To activate the PIN diodes $\:{D}_{A1}$ and $\:{D}_{B1}$, the DC voltage difference between $\:{V}_{1}$ and $\:{V}_{0}$ should be set to $\:1.0\text{V}$. Similarly to activate the PIN diodes $\:{D}_{A2}$ and $\:{D}_{B2}$ the DC voltage difference between $\:{V}_{2}$ and $\:{V}_{0}$ should be set to $\:1.0\text{V}$.

This vertical integration strategy enables a compact and planar realization of the antenna with minimal interference between the RF and DC domains, while maintaining the mechanical symmetry essential for polarization performance. The careful separation of functions across layers also facilitates easier fabrication, testing, and potential integration with multilayer RF front-end modules.

**Table 1:** Dimensions of the proposed reconfigurable circularly polarized antenna whose geometry is shown in (Fig. 1).

Parameter	$\:{D}_{H}$	$\:{L}_{A}$	$\:{L}_{B}$	$\:{L}_{C}$	$\:{L}_{D}$	$\:{L}_{E}$	$\:{L}_{F}$	$\:{L}_{G}$
Value (mm)	0.5	3.41	2.26	0.7	0.4	1.81	3.09	3.09
Parameter	$\:{L}_{H}$	$\:{L}_{P}$	$\:{L}_{S}$	$\:{R}_{C}$	$\:{W}_{C}$	$\:{W}_{F}$	$\:{W}_{G}$	$\:{W}_{T}$
Value (mm)	1.91	2.83	12	4.46	0.175	0.63	0.08	0.08

### Modeling PIN diodes for electromagnetic simulation

The reconfigurable functionality of the proposed antenna is enabled using M/A-COM’s M9AGP907 PIN diodes. These devices are based on Aluminum Gallium Arsenide (AlGaAs) flip-chip technology and are fabricated on OMCVD-grown epitaxial wafers. The fabrication process ensures high device uniformity and exceptionally low parasitic elements, making these diodes well-suited for high-frequency applications. The M9AGP907 diodes exhibit an extremely low RC product of approximately 0.1 ps and fast switching times on the order of 2–3 ns. The diodes are fully passivated with a silicon nitride layer and further protected with a polymer coating to prevent mechanical damage during assembly, particularly to the junction and anode air-bridge. Due to their ultra-low junction capacitance, these diodes are applicable up to millimeter-wave frequencies, especially in RF switches and switched phase shifter circuits. Their fast switching behavior and low parasitic loading make them ideal for pulsed and continuous-wave (CW) applications, including surface-mount multi-throw microwave switch assemblies, where minimal off-state loading is critical to preserving impedance matching and VSWR performance.

In the electromagnetic simulations, the PIN diodes were modeled in CST as lumped RLC elements bridging the discontinuities in the microstrip feed branches with the equivalent impedance values given by expressions (3) and (4). In the ON state, the diode was represented by a series resistor and inductor ($\:{R}_{S}=3\:{\Omega\:}$, $\:{L}_{P}\:=0.6\:\text{n}\text{H}$), while in the OFF state, it was modeled as a high-value resistor in parallel with a junction capacitor ($\:{R}_{P}=10\:\text{k}{\Omega\:}$, $\:{C}_{j}\:=0.15\:\text{p}\text{F}$). This simplified equivalent circuit effectively captures the frequency-dependent parasitic behavior of the PIN diodes, enabling accurate simulation of their impact on antenna performance at millimeter-wave frequencies. For M9AGP907 PIN diodes, the typical forward bias voltage at 10 mA is 0.95 V; the maximum forward bias voltage at 10 mA is 1.1 V. These values are typical for PIN diodes and are used during RF switching or biasing operations.

### Progressive design stages

The proposed reconfigurable circularly polarized antenna was developed through a series of systematic refinements, each targeting impedance matching, circular polarization (CP) quality, and polarization reconfigurability. Figure 2 illustrates the evolution from a basic notched patch to the final dual-branch feed configuration.

#### Stage 1 – basic notched patch (Fig. 3a)

**Fig. 4:**
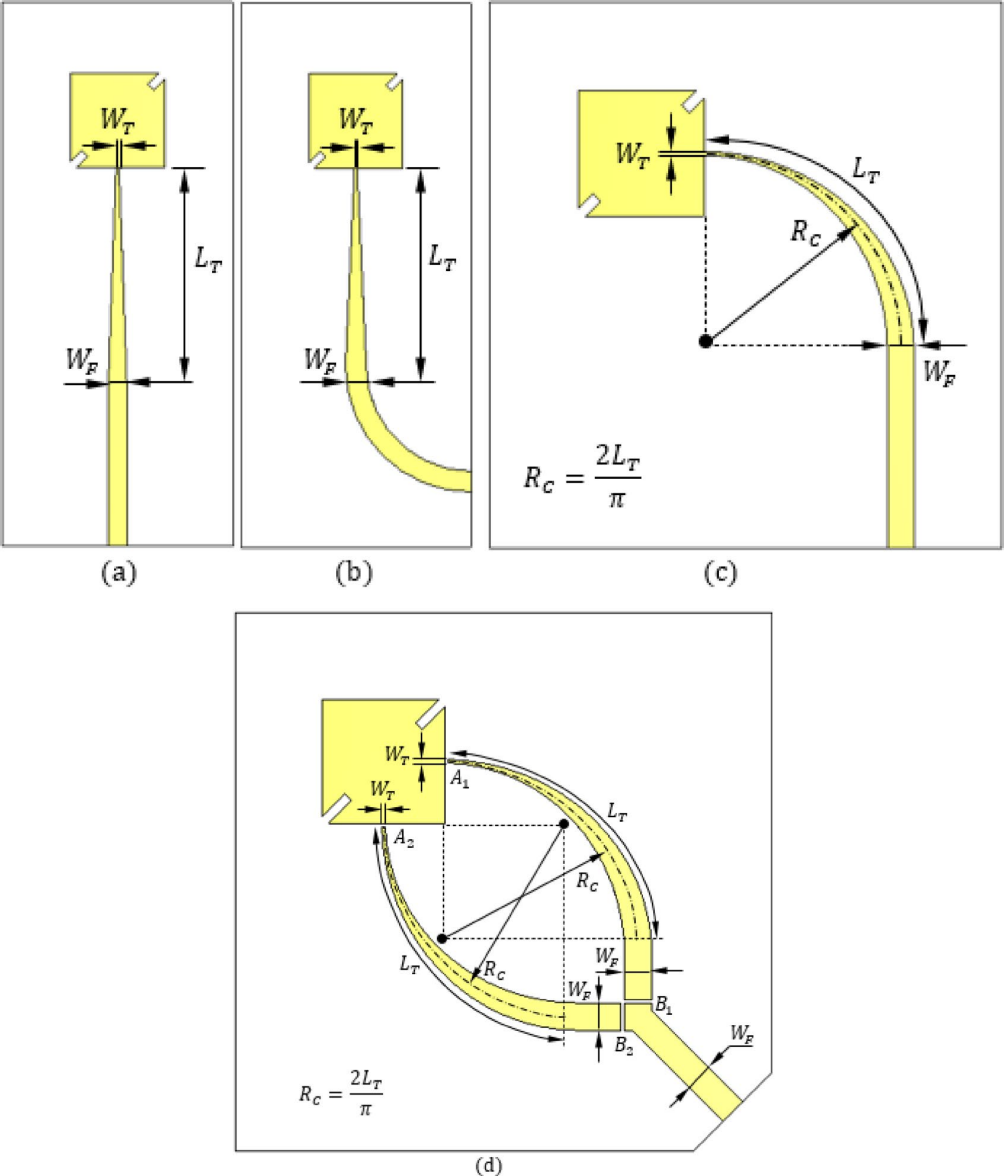
Progressive stages of the geometrical design of the proposed reconfigurable circularly polarized antenna. (**a**) Antenna with straight feed line. (**b**) Antenna with feed line of straight tapered region and curved uniform region. (**c**) Antenna with feed line of curved tapered region and straight unform region. (**d**) Antenna with feed line of branched geometry to allow LHCP by bridging the gaps “A1” and “B1” or RHCP by bridging the gaps “A2” and “B2”. The remaining dimensions are indicated in (Fig. 1; Table 1).

As shown in Fig. 4(a) A square patch with two diagonal notches is fed by a straight microstrip line. The notches perturb the orthogonal modes ($\:{TM}_{10}$​, $\:{TM}_{01}$​) to generate CP at 28 GHz. A tapered transition between the uniform feed and patch enhances impedance matching. Unlike an inset feed, this taper preserves patch symmetry about the 45° diagonal, which is critical for balanced mode excitation and stable CP performance.

#### Stage 2 – curved feed routing

To improve spatial routing, the feed is modified by curving the uniform-width section as shown in (Fig. 4b) while keeping the tapered portion straight at the patch edge. This geometry improves layout flexibility, maintains impedance characteristics, and preserves symmetry for reliable CP excitation.

#### Stage 3 – curved taper

Figure 4c shows an alternative way of spatial routing of the feed line. The tapered section itself is curved while the segment near the feed port remains straight. This layout enhances current distribution and further optimizes spatial routing. More importantly, it is better suited for integration with the dual-branch reconfigurable feed required for polarization switching.

#### Stage 4 – dual-branch reconfigurable feed

Figure 4d shows the final design where the main feed line is split into two symmetrical branches, each consisting of a curved taper and a uniform straight section. Gaps labeled A1–B1 (Branch 1) and A2–B2 (Branch 2) accommodate PIN diodes. By biasing the diodes selectively, either Branch 1 (for LHCP) or Branch 2 (for RHCP) is activated, exciting opposite patch edges and reversing the sense of polarization.

Through this stepwise evolution, the antenna maintains impedance matching, preserves modal symmetry for high CP purity, and introduces an effective, compact mechanism for electronic polarization reconfigurability, making it well-suited for dynamic mm-wave communication systems.

### Parametric study for enhanced antenna performance

The optimized values of the design parameters listed in Table 1 have been determined through an extensive parametric study aimed at maximizing the antenna’s performance. This study involved systematic electromagnetic simulations in which the dimensional parameters of both the radiating patch and the defected ground structure were varied. The objective was to evaluate their influence on the antenna’s reflection coefficient magnitude $\:\left|{S}_{11}\right|$ and axial ratio (AR), with the goal of achieving optimal impedance matching and circular polarization behavior.

**Fig. 5:**
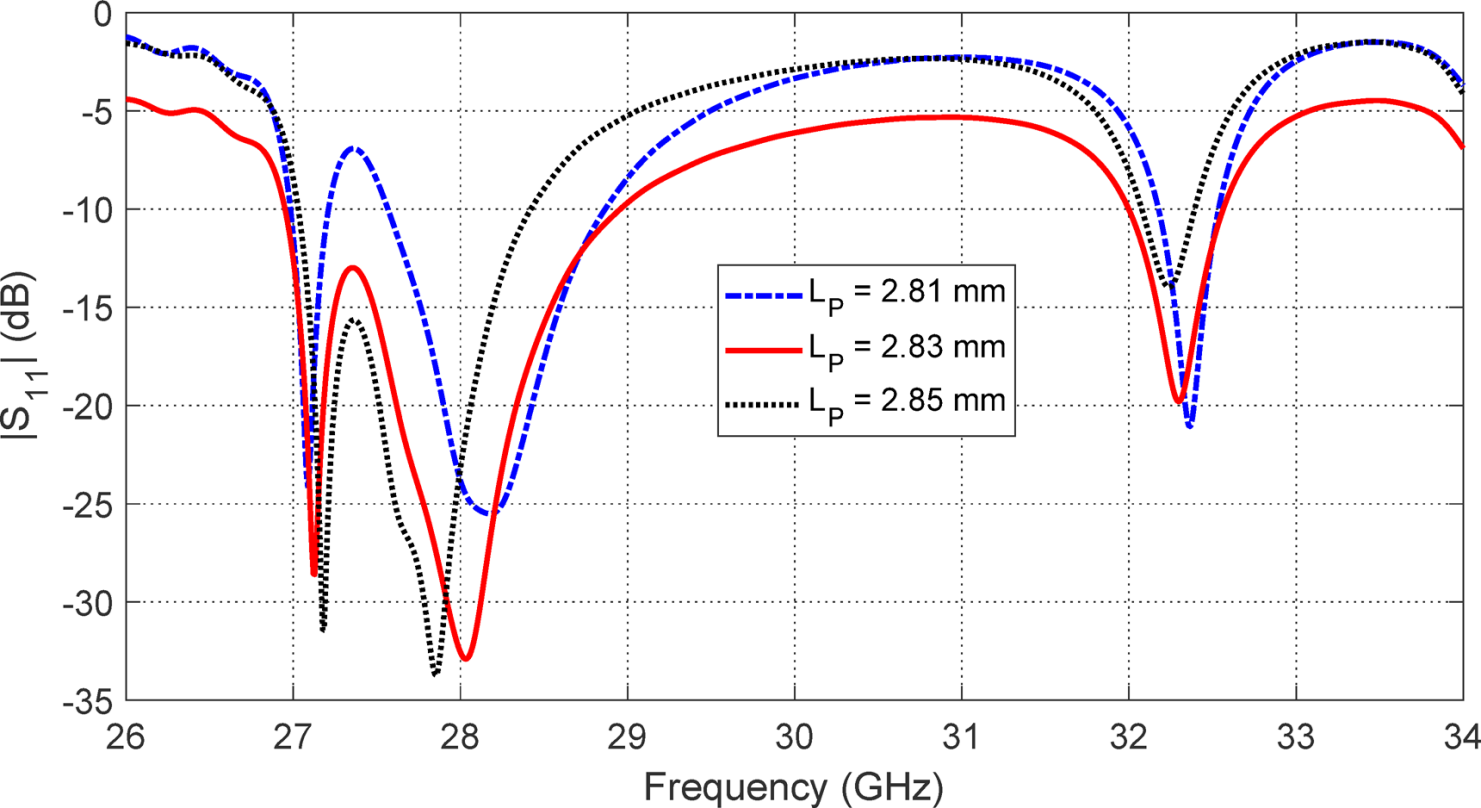
Variation of the frequency response of $\:\left|{S}_{11}\right|$ with varying the side length of the square patch, $\:{L}_{P}$.

**Fig. 6:**
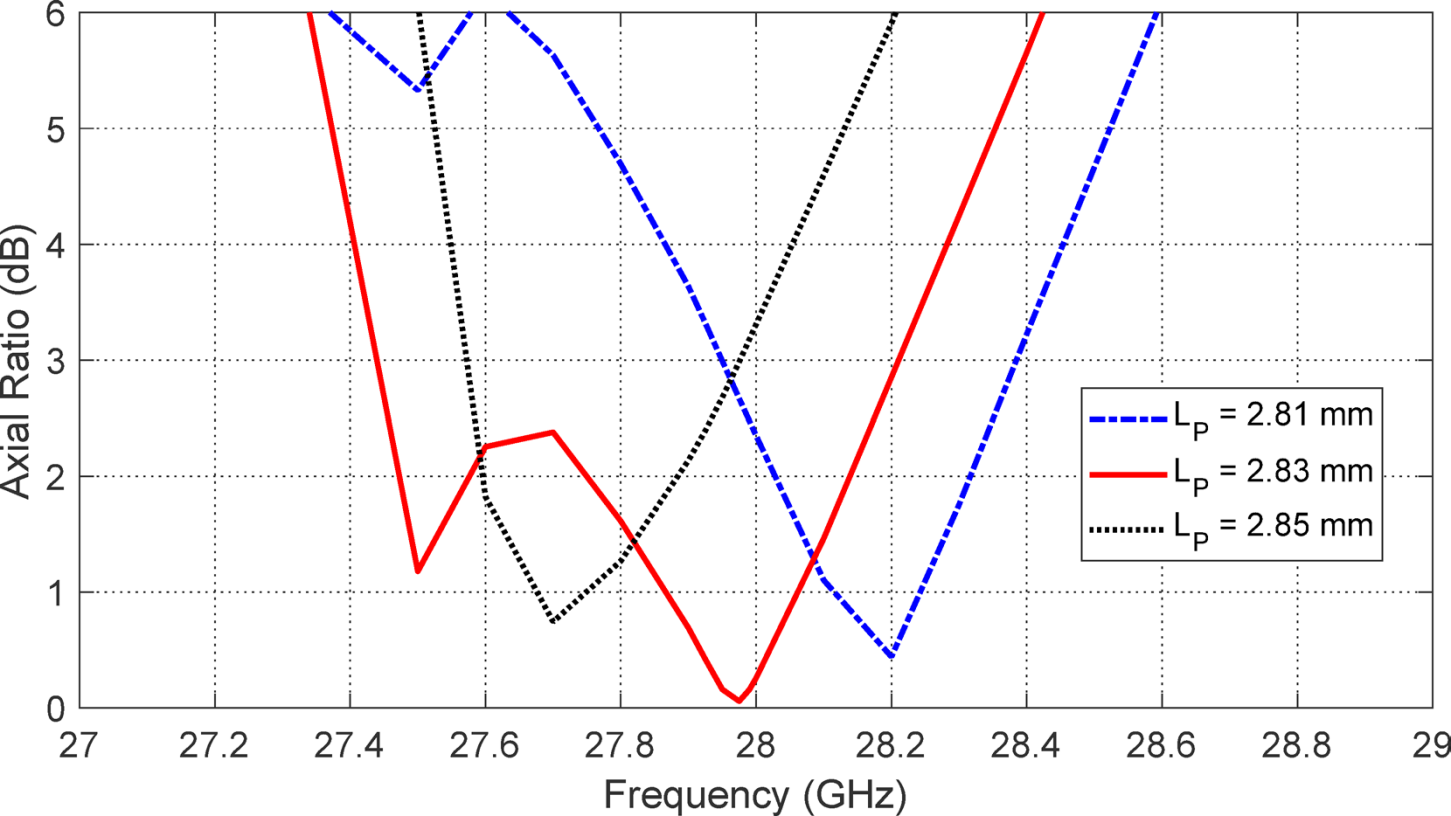
Variation of the frequency response of the axial ratio with varying the side length of the square patch, $\:{L}_{P}$.

**Fig. 7:**
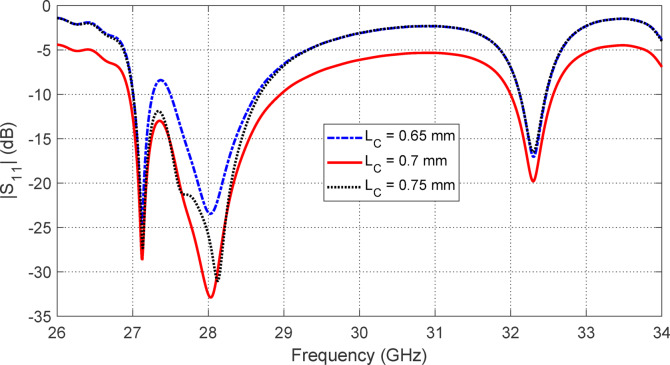
Variation of the frequency response of $\:\left|{S}_{11}\right|$ with varying the length of the notch at the corners of the square patch, $\:{L}_{C}$.

**Fig. 8:**
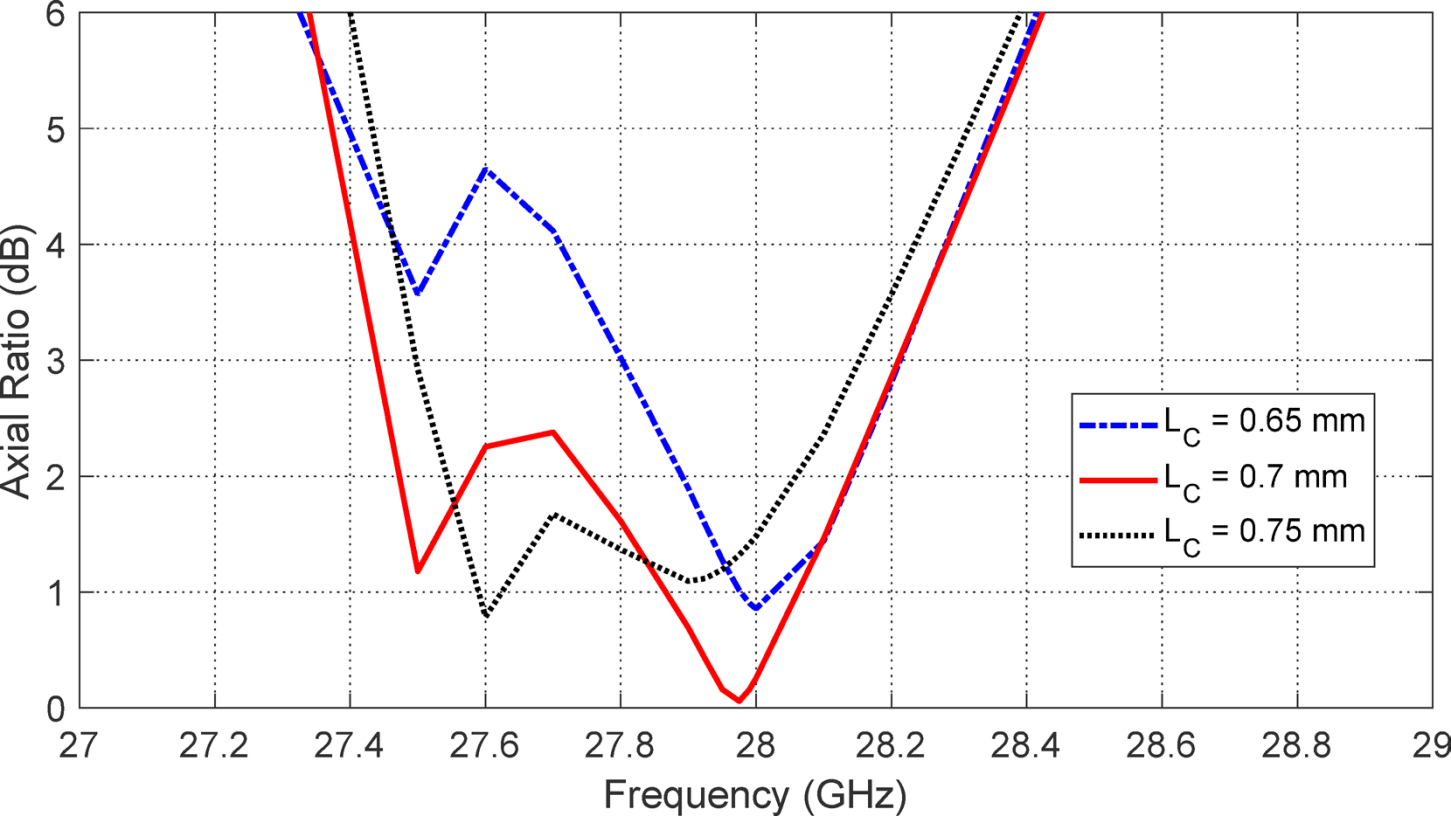
Variation of the frequency response of the axial ratio with varying the length of the notch at the corners of the square patch, $\:{L}_{C}$.

**Fig. 9:**
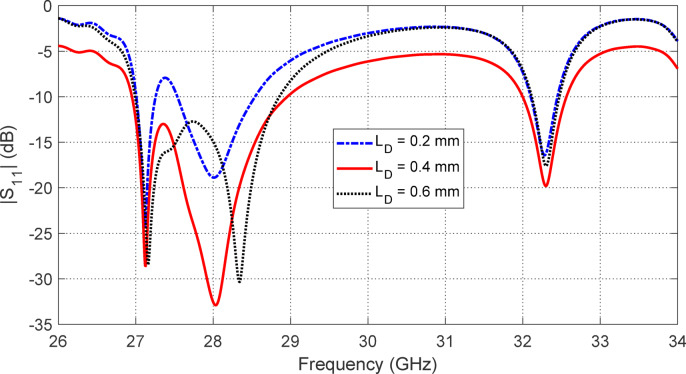
Variation of the frequency response of $\:\left|{S}_{11}\right|$ with varying the side length of the square aperture in the ground plane, $\:{L}_{D}$.

**Fig. 10:**
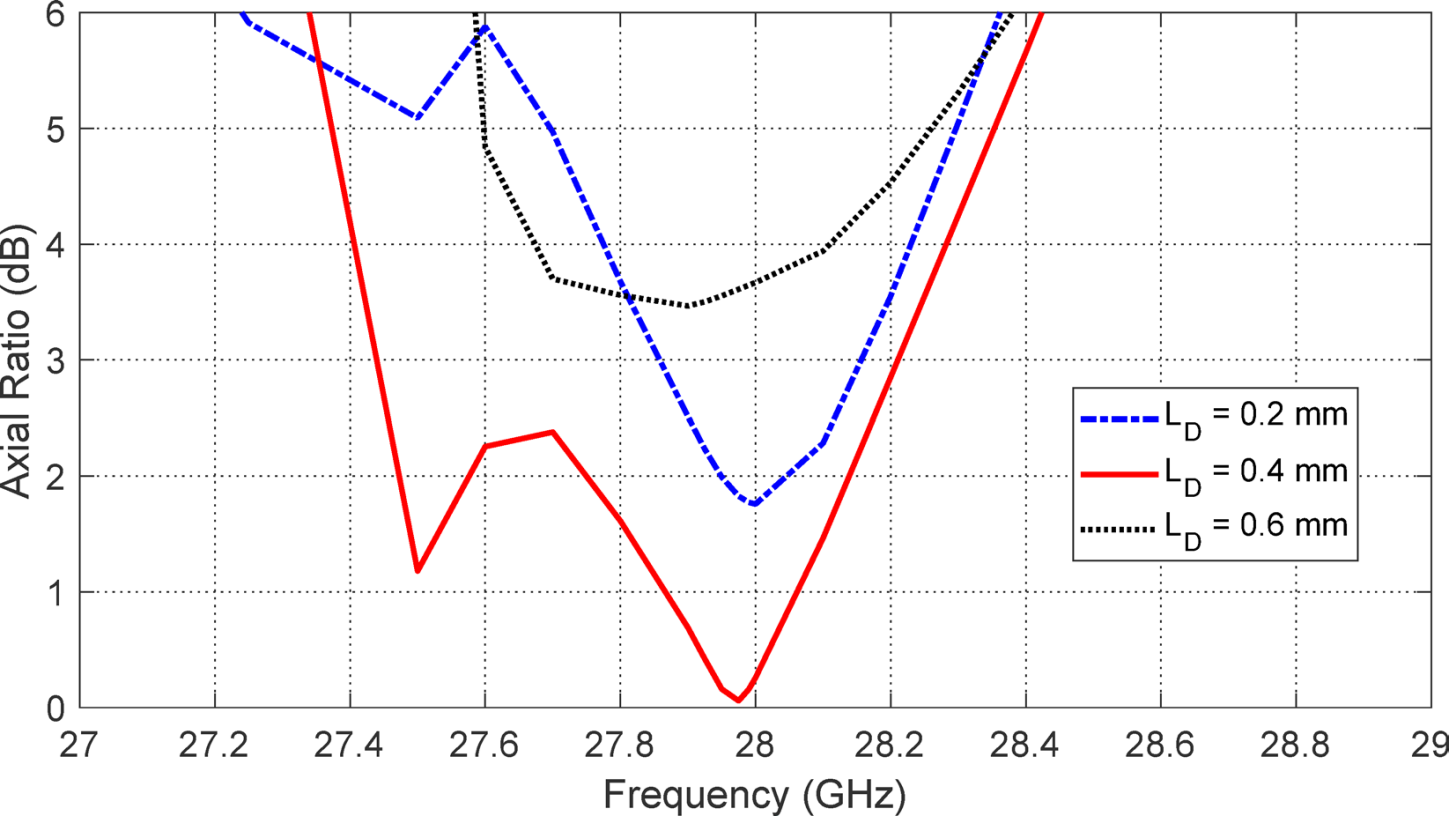
Variation of the frequency response of the axial ratio with varying the side length of the square aperture in the ground plane, $\:{L}_{D}$.

Figures 5, 6, 7, 8, 9 and 10 present the simulated responses illustrating how variations in key design parameters affect $\:\left|{S}_{11}\right|$ across the $\:26\--34\:\text{G}\text{H}\text{z}$ range and the axial ratio across the $\:27\--29\:\text{G}\text{H}\text{z}\:$range. The investigated parameters include $\:{L}_{P}$ (the side length of the square radiating patch) (see Figs. 5 and 6), $\:{L}_{C}$ (the length of the rectangular notches etched at the patch corners) (see Figs. 7 and 8), and $\:{L}_{D}$ (the side length of the square apertures in the ground plane located beneath the notched corners) (see Figs. 9 and 10). The results of the parametric sweeps indicate that the best overall antenna performance is achieved when the parameters are set to $\:{L}_{P}=2.83\:\text{m}\text{m}$, $\:{L}_{C}=0.7\:\text{m}\text{m}$, and $\:{L}_{D}=0.4\:\text{m}\text{m}$. Under these conditions, the antenna exhibits two well-defined impedance-matched bands: a lower band from $\:27$ to $\:29\:\text{G}\text{H}\text{z}$, and a higher band from $\:32$ to $\:32.6\:\text{G}\text{H}\text{z}$. Moreover, the axial ratio analysis confirms that the antenna achieves circular polarization within an $\:800\:\text{M}\text{H}\text{z}$ bandwidth, extending from $\:27.42$ to $\:28.22\:\text{G}\text{H}\text{z}$, where the axial ratio remains below the $\:3\:\text{d}\text{B}$ threshold. This frequency range corresponds to the lower operational band and confirms the effectiveness of the proposed geometry in supporting circular polarization through careful tuning of the structural parameters.

### Novelty aspects of the proposed reconfigurable CP antenna

Unlike previously reported polarization-reconfigurable antennas that depend on dual-port excitation using external 90° hybrid couplers or mechanically actuated components, the proposed design introduces a highly compact, fully electronically reconfigurable circularly polarized (CP) antenna operating in the millimeter-wave band. Polarization switching is achieved entirely through a single-port microstrip feed network, incorporating six PIN diodes arranged in a branched topology. This configuration enables polarization control without external RF components, thereby reducing system complexity, insertion loss, and footprint while maintaining stable CP performance.

The key novelty aspects of the proposed design are as follows: (i) *Simplified Radiator Geometry (Notched Square Patch)*: The antenna employs notched square patch geometry, avoiding intricate structures such as multi-arm slots, metasurfaces, or complex fractals. Despite its simplicity, the radiator effectively supports CP operation and reconfigurability. (ii) *Low-Loss*,* High-Quality Substrate (Rogers RO3003)*: Fabrication on a low-loss RO3003 substrate (dielectric constant $\:{\epsilon\:}_{r}$ = 3.0, loss tangent, $\:\text{tan}\delta\:\:=\:0.0013$) enhances the antenna’s efficiency and minimizes dielectric losses, which are critical at millimeter-wave frequencies. (iii) *Compact and Efficient Reconfigurable Feed Network*: The single-feed microstrip layout directly selects which patch edge is excited through diode-controlled branches, eliminating the need for external hybrid couplers or additional feed ports. This contributes to a reduced footprint, lower RF loss, and simplified biasing and integration with transceivers. (iv) *Use of Ultra-Low-Loss PIN Diodes*:

The M9AGP907 AlGaAs PIN diodes feature extremely low ON-state resistance and minimal junction capacitance. Their inclusion minimizes impedance mismatch and signal attenuation in the reconfigurable network, ensuring reliable switching and stable performance across the operating band. (v) *Full CP Sense Reconfigurability via Single-Port Excitation*: Unlike dual-port CP antennas that rely on external circuitry to alter the polarization state, this design achieves left-hand and right-hand CP sense switching via simple electronic control of the diode states—while maintaining a single RF input port. This is a highly practical solution for compact and integrated transceiver systems. (vi) *Robust Performance in the Millimeter-Wave Band*: Achieving polarization reconfigurability at millimeter-wave frequencies presents unique challenges due to diode parasitics, increased conductor losses, and layout sensitivity. The proposed antenna overcomes these challenges through careful design and optimization, validated through full-wave simulation and hardware prototyping. (vii) *Fine-Resolution Prototyping Using LPKF Milling*:

The prototype was fabricated using a high-precision LPKF Prototyping Machine, enabling sub-$\:100\:{\upmu\:}\text{m}$ resolution in trace and gap dimensions. This level of precision is essential to accurately implement the compact reconfigurable feed network and maintain performance integrity at millimeter-wave frequencies.

## Experimental work

To validate the simulated performance of the proposed reconfigurable antenna, a prototype was fabricated and experimentally characterized. The evaluation involved measuring both the reflection coefficient and far-field radiation properties under different reconfiguration states. The results are compared with simulation data to verify the antenna’s practical performance and reconfigurability. The experimental process is described in the following subsections.

### Antenna fabrication

The fabrication of the proposed reconfigurable antenna requires high precision to realize the fine transmission lines, narrow gaps, and multilayer integration needed for millimeter-wave operation. To achieve this, a laser-based prototyping approach was adopted, using the LPKF ProtoLaser U4 system for patterning, drilling, and via formation. This technique enables sub-50 μm resolution and accurate alignment across layers, ensuring that the antenna geometry, defected ground structure, and biasing network are fabricated with minimal parasitics and excellent repeatability. The following subsections describe the fabrication tool, materials, and assembly procedure in detail.

#### Fabrication tool (LPKF machine)

**Fig. 11:**
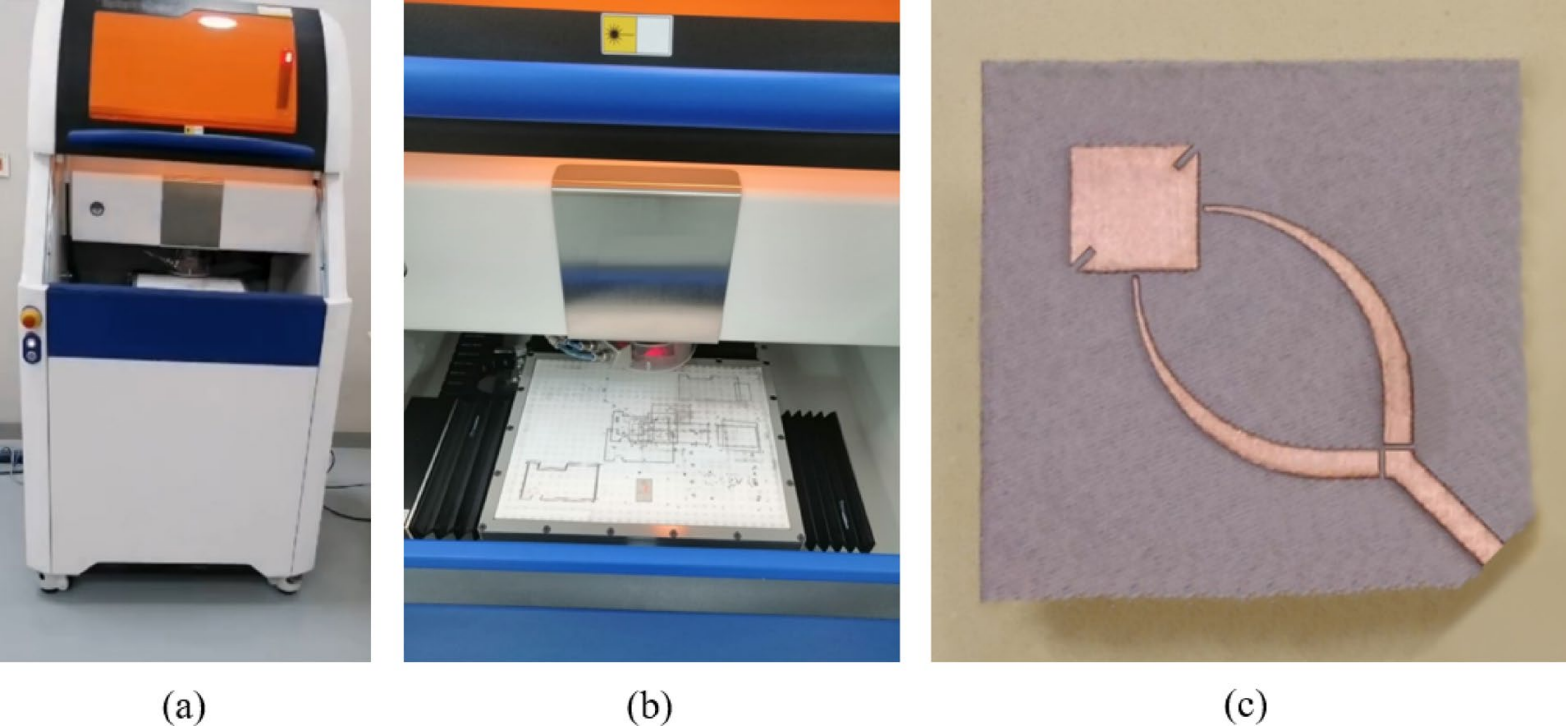
The LPKF ProtoLaser U4 machine used in this work to fabricate the proposed antenna. (**a**) The LPK machine. (**b**) Zoomed view at the working area. (**c**) The printed antenna.

The LPKF ProtoLaser U4 system, shown in Fig. 11, was employed to fabricate the proposed multilayer reconfigurable antenna. It is a laser-based PCB prototyping platform designed for high-precision microwave and millimeter-wave circuit fabrication. The system uses a finely focused ultraviolet laser beam to selectively ablate copper, enabling direct patterning of conductive traces without requiring chemical etching. This non-contact laser structuring ensures minimal thermal stress on the substrate, preserving its dielectric properties.

The ProtoLaser U4 provides a minimum track width of approximately 50 μm and minimum spacing of 13 μm, achievable with a ~ 20 μm laser spot size. These capabilities are essential for fabricating the fine transmission lines, narrow gaps, and accurate slot geometries required for reconfigurable antenna designs operating in the mm-wave band. In addition to copper removal, the machine performs high-precision drilling of microvias and through-holes, which are used for biasing networks and interlayer connections in multilayer layouts.

A key advantage of the system is its suitability for rapid prototyping and iterative optimization. By eliminating wet chemical processes, the LPKF machine ensures a clean, fast, and environmentally friendly fabrication process. Furthermore, its ability to achieve sub-50 μm precision enables reliable integration of multilayer structures, such as the antenna, defected ground plane, and biasing circuits, while minimizing misalignment and parasitic effects. These features make the LPKF ProtoLaser U4 particularly well-suited for the development of advanced high-frequency antenna prototypes.

#### Three layers fabrication process

The antenna is fabricated on Rogers RO3003 substrates, each with a thickness of $\:h=250\:{\upmu\:}\text{m}$. Both the radiating structure and the biasing network are implemented on identical RO3003 substrates to maintain consistent electrical and mechanical properties across all layers. The two substrates are bonded together using a very thin layer of adhesive material, forming an integrated double-substrate structure. In the resulting configuration, the top face accommodates the antenna along with its feeding network, while the bottom face hosts the biasing circuit.

To support high-frequency operation and ensure precise impedance matching, the narrower width of each tapered feed line branch is set to $\:{W}_{T}=80\:{\upmu\:}\text{m}$. The widest end of the tapered line, which connects to the uniform section of the feed, has a width of $\:{W}_{F}=630\:{\upmu\:}\text{m}$, corresponding to a characteristic impedance of 50 Ω. Additionally, the gaps introduced for PIN diode integration, which enable the selective activation of individual feed branches, are designed to be $\:{W}_{G}=80\:{\upmu\:}\text{m}$. This minimal gap width ensures that electromagnetic discontinuities are kept to a minimum while still allowing reliable electronic switching.

**Fig. 12:**
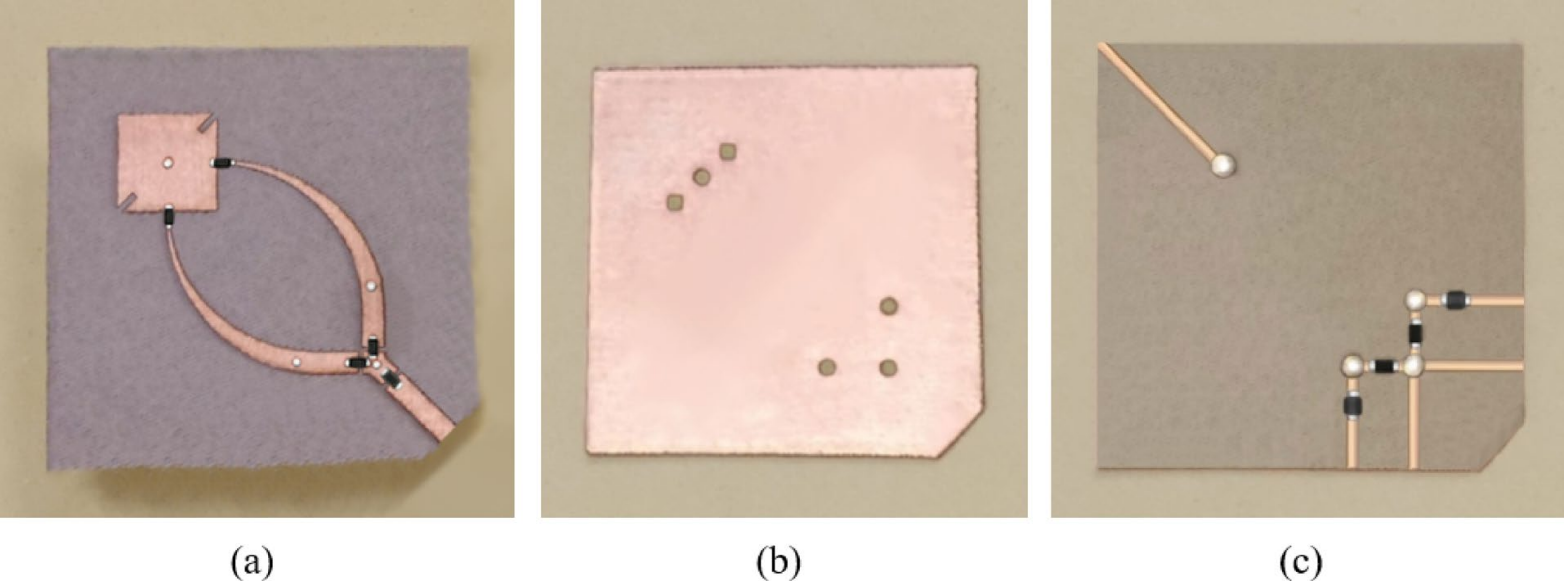
The fabricated three-layred antenna structure. (**a**) Top layer hosting the antenna, feed network, and PIN diodes. (**b**) Middle layer incorporating the DGS. (**c**) Bottom layer including the DC biasing network.

Due to the fine geometrical requirements of the design, the antenna is fabricated as a three-layer structure using a high-precision LPKF laser-based system, which provides minimum width of $\:50\:{\upmu\:}\text{m}$ and minimum spacing of $\:13\:{\upmu\:}\text{m}$. This level of accuracy ensures the reliable realization of narrow lines, precise gaps, and accurately aligned vias required by the multilayer configuration. The precision of the LPKF fabrication process is crucial for achieving consistent performance in the millimeter-wave frequency range. The three layers of the fabricated antenna are shown in (Fig. 12).

#### Assembly of the three-layer structure

The three layers illustrated in Fig. 12 were bonded together using a very thin adhesive film. The adhesive is nearly transparent at millimeter-wave frequencies, with a dielectric constant close to unity, ensuring negligible influence on the antenna’s electromagnetic performance. This bonding method provides reliable mechanical stability while preserving the intended impedance and radiation characteristics of the integrated structure.

### Measurement of the reflection coefficient

**Fig. 13:**
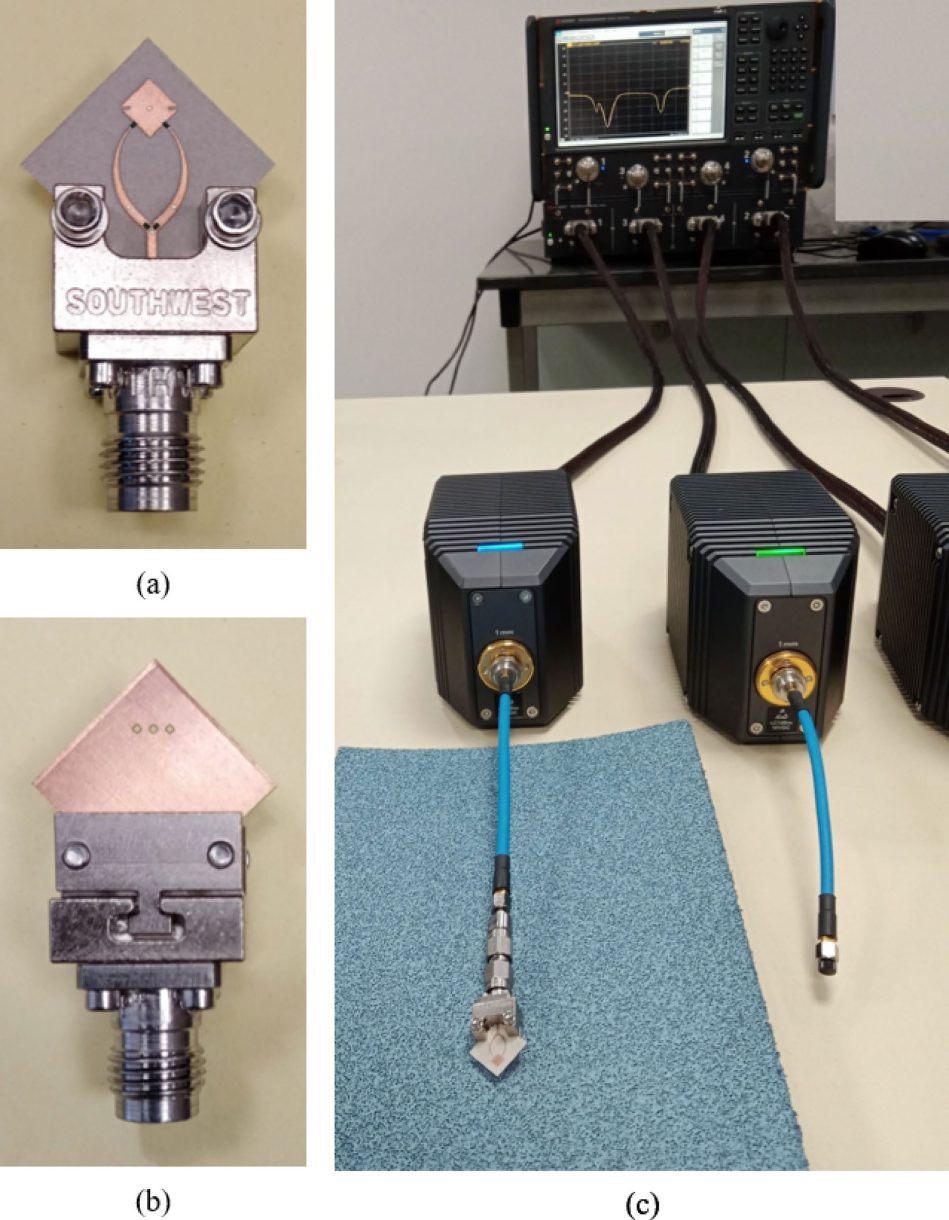
Experimental setup for measurinf the reflection coefficient $\:{S}_{11}$ at the feeding port of the proposed antenna. (**a**) Top view of the antenna mounted to the coaxial launcher. (**b**) Back view of the antenna mounted to the coaxial launcher.

The reflection coefficient of the proposed antenna is measured using a four-port Keysight N5244B PNA-X vector network analyzer (VNA), which covers a frequency range of 10 MHz to 43.5 GHz. To extend the measurement capability up to 110 GHz, four external frequency extender modules (model N5293AX03) are connected to the VNA ports, as shown in Fig. 13. This configuration enables full S-parameter characterization across the 10 MHz–110 GHz range and is suitable for both passive and active device measurements.

The N5244B system integrates dual internal signal sources, broadband S-parameter and noise receivers, internal pulse modulators, RF switches, signal combiners, and RF access points. This built-in functionality is particularly advantageous for evaluating reconfigurable antennas, such as the proposed design, which requires DC biasing of switching elements during operation.

As shown in Fig. 13a, the fabricated antenna is mounted using a Southwest Microwave 2.4 mm coaxial launcher, enabling wideband coaxial-to-microstrip transition. The launcher was connected to Port 1 of the VNA via the corresponding frequency extender, as illustrated in (Fig. 13b). The system was calibrated appropriately, and the reflection coefficient $\:{S}_{11}$​ was measured at the antenna’s feed port under real operating conditions, including DC biasing for polarization control.

The measured frequency response of $\:\left|{S}_{11}\right|$ indicates two distinct impedance-matched bands. The first band, centered at 28 GHz (from $\:27\:\text{G}\text{H}\text{z}$ to $\:29\:\text{G}\text{H}\text{z}$), corresponds to the antenna’s circularly polarized (CP) mode of operation. The second band, centered at $\:32.3\:\text{G}\text{H}\text{z}$ (from $\:32\:\text{G}\text{H}\text{z}\:\text{t}\text{o}\:32.6\:\text{G}\text{H}\text{z}$), corresponds to a linearly polarized (LP) mode. These measurements confirm the successful polarization reconfigurability of the antenna, as well as its effective impedance matching in both operational states across the targeted millimeter-wave frequency ranges.

**Fig. 14:**
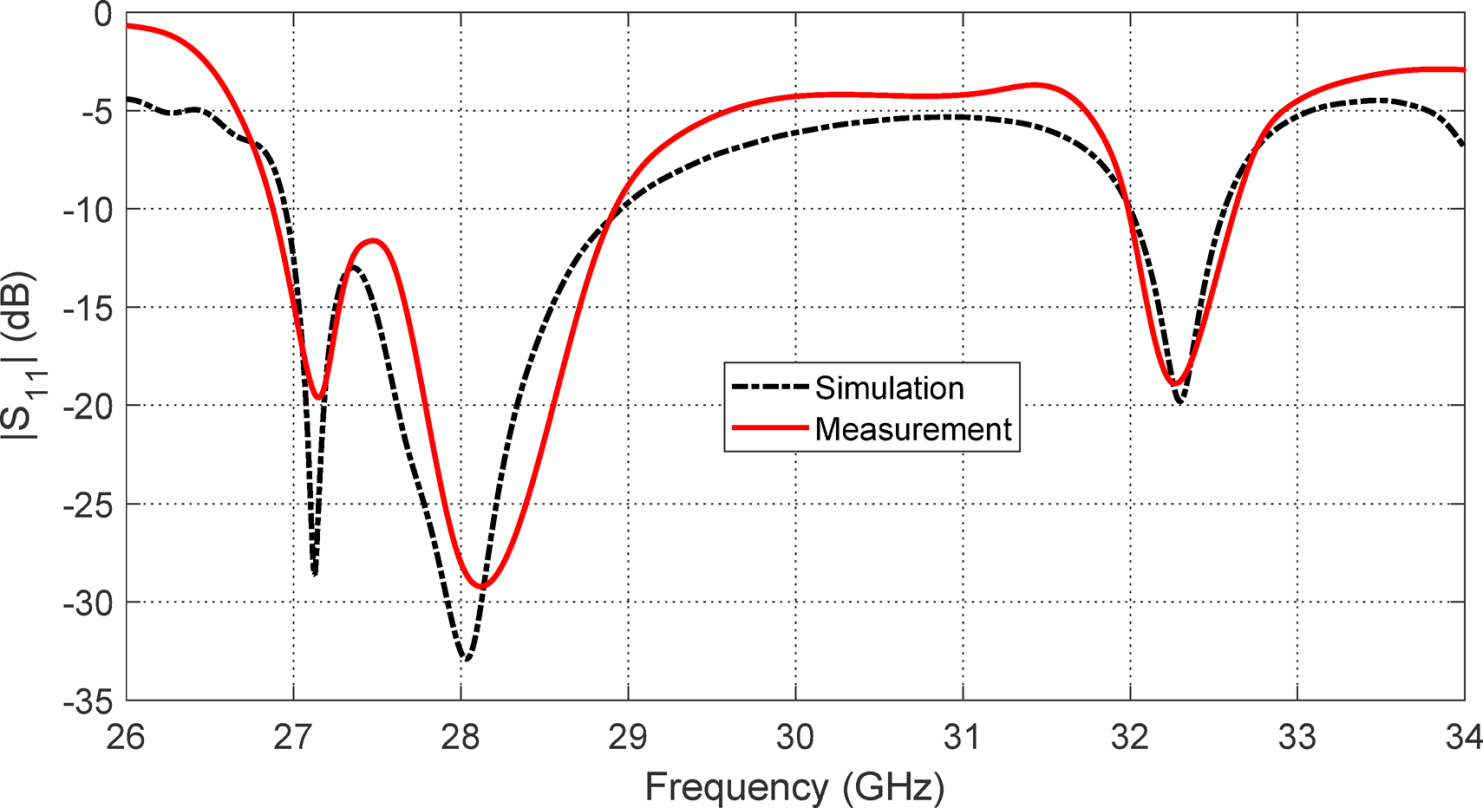
Comparison between the simulation and experimental results of the variation of the reflection coefficient magnitude $\:\left|{S}_{11}\right|$ of the proposed reconfigurable antenna.

Figure 14 presents a comparison between the simulated and measured reflection coefficient magnitude $\:\left|{S}_{11}\right|$ of the proposed reconfigurable antenna across the targeted frequency range. The reflection coefficient $\:\left|{S}_{11}\right|$ is a key performance metric that quantifies the impedance matching of the antenna; values below − 10 dB are typically indicative of good matching, enabling efficient power transfer between the antenna and the feeding network with minimal reflection.

Both simulation and measurement results clearly reveal the presence of two distinct impedance matching frequency bands. Specifically, the lower band spans from $\:27\:\text{G}\text{H}\text{z}$ to $\:29\:\text{G}\text{H}\text{z}$, while the higher band extends from $\:32\:\text{G}\text{H}\text{z}\:\text{t}\text{o}\:32.6\:\text{G}\text{H}\text{z}$. These two bands confirm the antenna’s reconfigurability and its ability to support multi-band operation, which is highly desirable in modern mm-wave systems for frequency agility and spectrum reuse.

The measured results show good agreement with the simulated predictions, reinforcing the accuracy of the antenna design and validating the physical realization. In both cases, the reflection coefficient falls well below the $\:-10\:\text{d}\text{B}$ threshold across the two bands, indicating robust impedance matching. The dual-band behavior is thus successfully achieved in practice, as intended by the design.

Minor deviations between the measured and simulated curves can be observed, such as slight frequency shifts or differences in the depth of the reflection minima. These are commonly attributed to practical limitations such as fabrication tolerances, soldering quality, connector mismatches, and uncertainties in the measurement setup (e.g., calibration errors, cable effects, and connector repeatability). Nevertheless, these discrepancies are relatively small and do not compromise the antenna’s operational performance.

Overall, Fig. 14 confirms that the proposed antenna meets its design goals of achieving dual-band impedance matching around $\:28\:\text{G}\text{H}\text{z}$ and $\:32\:\text{G}\text{H}\text{z}$. The close agreement between simulated and measured $\:\left|{S}_{11}\right|$ validates both the design methodology and fabrication process, demonstrating the antenna’s suitability for mm-wave communication applications where reliable dual-band performance is critical.

### Far field measurement

**Fig. 15:**
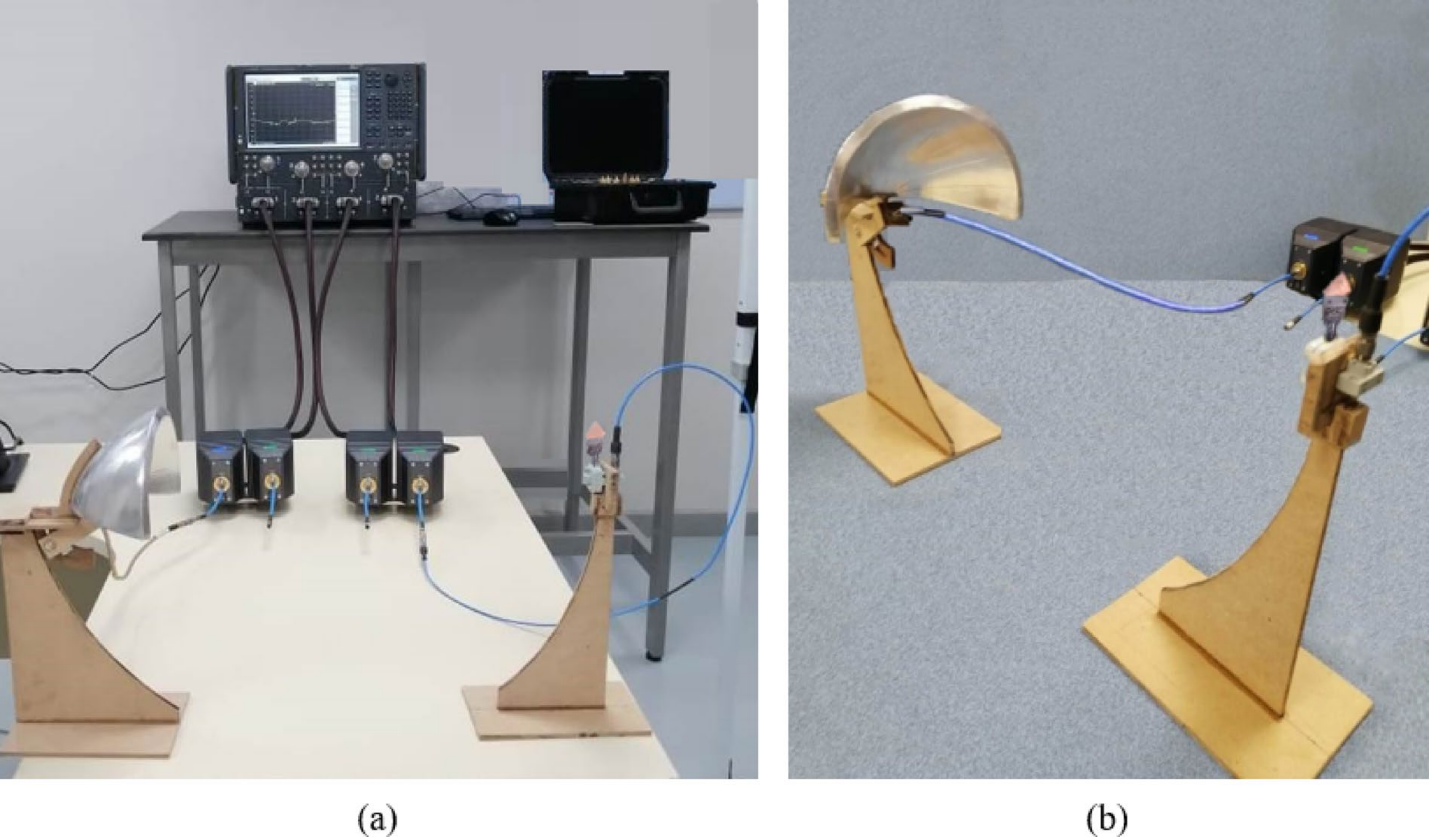
Measurement of the gain, axial ratio, and radiation pattern of the proposed antenna. (**a**) Experimental setup of the compact antenna test range, illustrating the equipment and connections used for far-field characterization. The system includes a VNA, frequency extender modules, and control hardware. (**b**) Photograph of the antenna under test (AUT) and the offset parabolic reflector placed inside an anechoic chamber, where the inner walls are coated with flat absorbers optimized for mm-wave measurements.

Figure 15 provides a comprehensive visual representation of the far-field measurement setup used to evaluate the radiation characteristics of the proposed reconfigurable circularly polarized (CP) antenna. Subfigure 15a demonstrates the complete measurement setup, highlighting the system’s modularity and suitability for high-frequency characterization. This is a compact antenna test range (CATR) using the offset feed parabolic reflector as a source of quasi-plane waves. The inclusion of frequency extenders allows the Keysight N5244B VNA to measure up to 110 GHz, covering both the CP and LP bands of the antenna. Subfigure 15(b) shows the antenna under test and the offset parabolic reflector placed in a controlled anechoic environment. The flat absorbers lining the chamber reduce unwanted reflections and simulate free-space conditions, ensuring accurate far-field radiation measurements. The location of the frequency extenders inside the chamber reduces signal path length and minimizes cable losses at mm-wave frequencies, while the VNA is left outside for accessibility and thermal stability.

Importantly, the measurement approach is based on capturing the transmission coefficient $\:{S}_{21}$​ between the AUT and the reflector feed, which directly reflects the antenna’s radiation behavior in different directions. The measured results indicate that the antenna achieves circular polarization within the $\:27.7\--28.5\:\text{G}\text{H}\text{z}$ range (as verified by an axial ratio below $\:3\:\text{d}\text{B}$), and supports linear polarization within the $\:32.8\--33.2\:\text{G}\text{H}\text{z}$ band. This confirms the antenna’s dual-mode operation and validates the effectiveness of the reconfigurable feed structure.

#### Experimental results for the radiation pattern

**Fig. 16:**
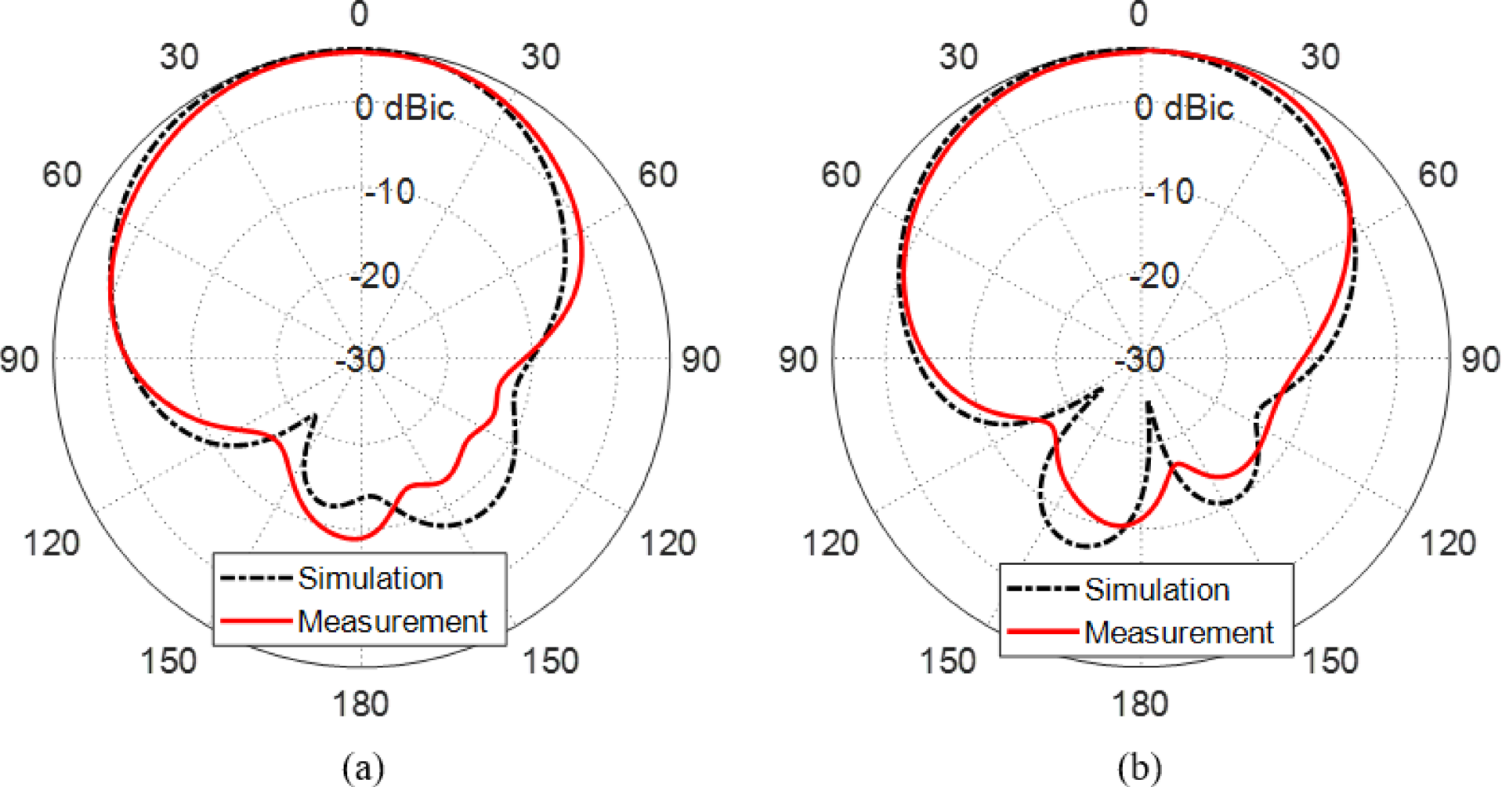
Comparison between the LHCP gain patterns at $\:28\:\text{G}\text{H}\text{z}$ obtained by simulation and measurement for the proposed reconfigurable antenna when the PIN diodes $\:{D}_{A1}$ and $\:{D}_{B1}$ are activated (forward biased) whereas the PIN diodes $\:{D}_{A2}$ and $\:{D}_{B2}$ are deactivated. (**a**) Plane $\:\varphi\:=0^\circ\:$ (**b**) Plane $\:\varphi\:=90^\circ\:$.

Figure 16 illustrates a comparison between the simulated and measured LHCP gain radiation patterns of the proposed reconfigurable antenna in two principal planes: (a) ϕ = 0° and (b) ϕ = 90°, under the configuration where PIN diodes $\:{D}_{A1}$ and $\:{D}_{B1}$ are activated (forward-biased), and $\:{D}_{A2}$ and $\:{D}_{B2}$ are deactivated (reverse-biased). This diode configuration enables the antenna to operate in its LHCP radiation mode centered around $\:28\:\text{G}\text{H}\text{z}$.

From both simulation and measurement, the gain patterns demonstrate a well-defined directional radiation behavior characteristic of a circularly polarized antenna. The measured LHCP gain peaks at approximately $\:6.2\:\text{d}\text{B}\text{i}\text{c}$, which aligns closely with the simulated result, validating the efficiency of the radiation mechanism and the accuracy of the design.

In both planes, the radiation patterns preserve a consistent main lobe direction, and the beam shapes remain largely symmetrical, indicating stable polarization purity and structural balance in the physical prototype. Minor discrepancies between the measured and simulated patterns are evident, particularly in side lobe levels and slight angular shifts of the peak gain direction. These differences are typical in practical measurements and can be attributed to manufacturing imperfections, substrate tolerances, soldering of surface-mount components, and uncertainties in the anechoic chamber measurement setup.

Importantly, the results confirm that the antenna maintains its reconfigurability and polarization switching capability, as the activation of $\:{D}_{A1}$ and $\:{D}_{B1}$ successfully establishes LHCP radiation with substantial gain. The presence of well-matched gain patterns in both planes also suggests good cross-polarization suppression and stable performance across different angular directions.

Overall, Fig. 16 confirms that the proposed antenna achieves effective LHCP operation with a peak gain of $\:6.2\:\text{d}\text{B}\text{i}\text{c}$ and consistent radiation characteristics in both principal planes, demonstrating its suitability for mm-wave communication systems requiring polarization diversity and beam pattern stability.

**Fig. 17:**
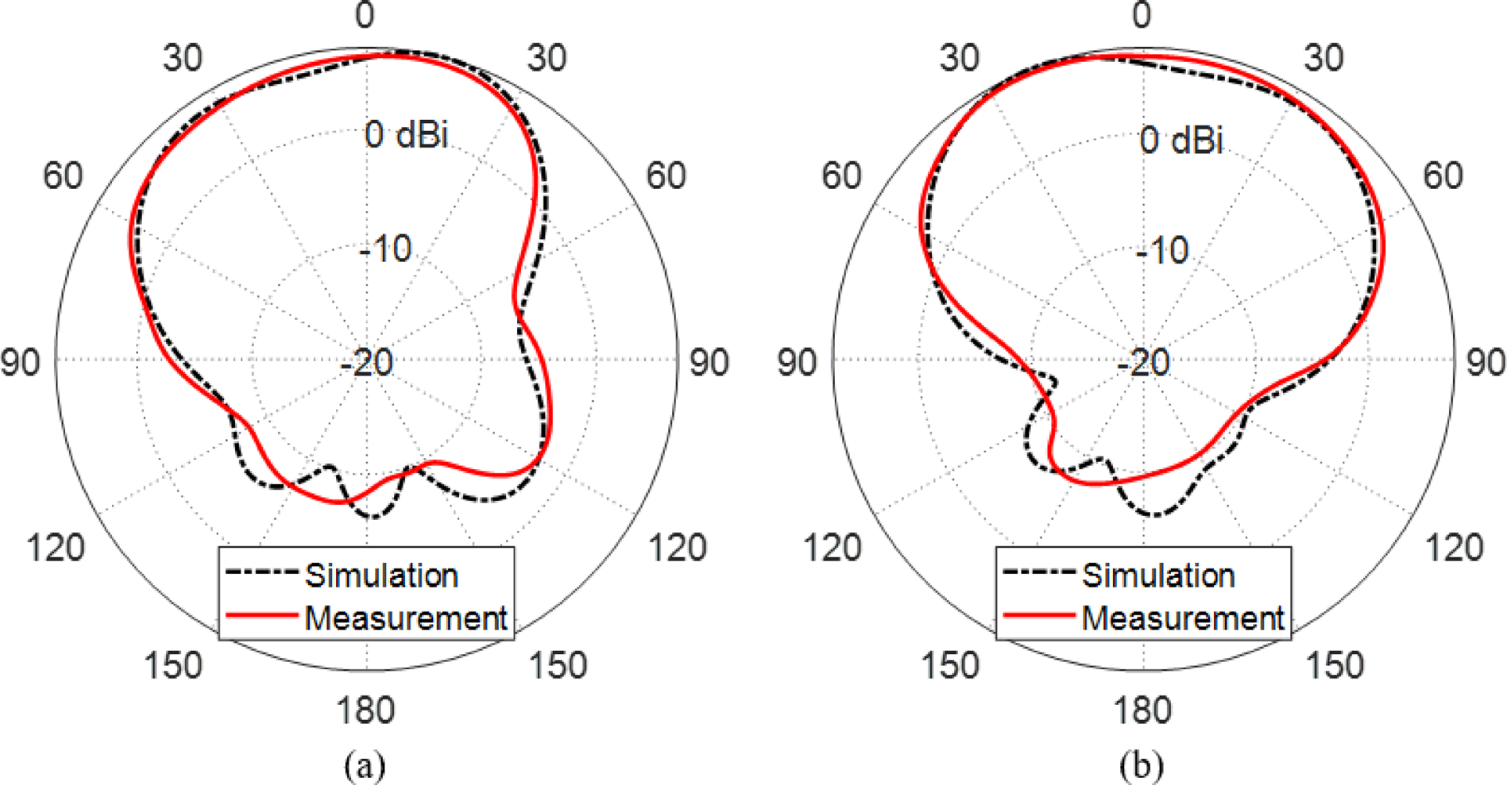
Comparison between the gain patterns at 32.3 $\:\text{G}\text{H}\text{z}$ obtained by simulation and measurement for the proposed reconfigurable antenna when the PIN dioes $\:{D}_{A1}$ and $\:{D}_{B1}$ are activated whereas the PIN diodes $\:{D}_{A2}$ and $\:{D}_{B2}$ are deactivated. (**a**) Plane $\:\varphi\:=0^\circ\:$ (**b**) Plane $\:\varphi\:=90^\circ\:$.

Figure 17 compares the simulated and measured gain patterns at 32 GHz for the proposed reconfigurable antenna under a specific switching condition: PIN diodes $\:{D}_{A1}$ and $\:{D}_{B1}$ are activated, while $\:{D}_{A2}$ and $\:{D}_{B2}$ are deactivated. This diode configuration results in a linearly polarized radiation mode, distinct from the circularly polarized mode observed under other biasing conditions. Both subfigures (a) and (b) illustrate the radiation behavior in two principal planes, $\:\varphi\:\:=\:0^\circ\:$ (E-plane) and $\:\varphi\:\:=\:90^\circ\:\:$(H-plane). The antenna exhibits stable and symmetrical radiation patterns with good agreement between simulation and measurement in both planes, demonstrating consistent directivity and beam shape across the test setup. The antenna achieves linear polarization over the frequency band $\:32\:\--\:32.6\:\text{G}\text{H}\text{z}$, which corresponds to one of its two impedance-matching bands. Within this band, the antenna exhibits a maximum realized gain of approximately $\:7.2\:\text{d}\text{B}\text{i}$, indicating strong directional radiation suitable for high-frequency point-to-point millimeter-wave communication links. The measured patterns closely follow the simulated ones, confirming the design’s accuracy and the effectiveness of the reconfigurable architecture. The main lobes in both planes are well-defined and aligned, while the sidelobe levels and back radiation remain sufficiently low, contributing to the high radiation efficiency and pattern stability.

The results presented in Fig. 17 confirm that the antenna not only supports frequency and polarization reconfiguration but also maintains high-gain and clean radiation patterns in both circularly and linearly polarized modes. This enhances its versatility for diverse mm-wave applications, such as adaptive beam steering, polarization diversity, and dynamic channel selection.

**Fig. 18:**
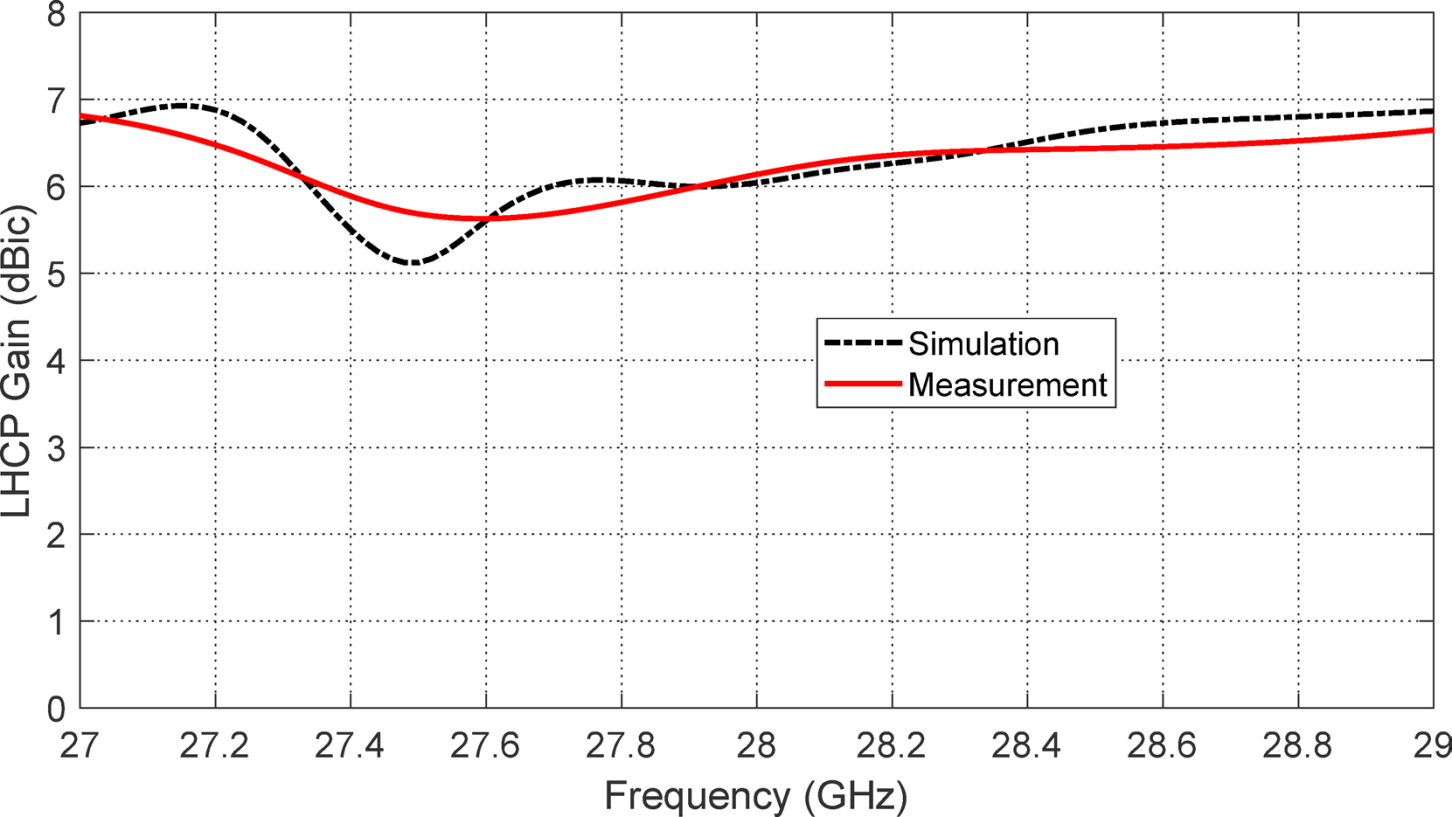
Comparison between the simulation and experimental results for the frequency response of the LHCP gain of the proposed reconfigurable antenna over the frequency range $\:27-29\:\text{G}\text{H}\text{z}$ (lower frequency band of operation) when the PIN diodes $\:{D}_{A1}$ and $\:{D}_{B1}$ are activated (forward biased) whereas the PIN diodes $\:{D}_{A2}$ and $\:{D}_{B2}$ are deactivated. The LHCP gain is $\:6.2\:\text{d}\text{B}\text{i}\text{c}$ at $\:28\:\text{G}\text{H}\text{z}$.

Figure 18 presents a comparison between the simulated and measured LHCP gain of the proposed reconfigurable antenna across the frequency range 27–29 GHz, which corresponds to the antenna’s lower operating band. This frequency band is specifically designed for circular polarization, achieved when the PIN diodes $\:{D}_{A1}$ and $\:{D}_{B1}$ are activated and $\:{D}_{A2}$ and $\:{D}_{B2}$ are deactivated (reverse biased). This diode configuration enables the antenna to support circularly polarized radiation, optimized near $\:28\:\text{G}\text{H}\text{z}$. The figure shows good agreement between simulation and experimental results, validating the effectiveness of the antenna design and the reliability of both fabrication and measurement processes. Both curves exhibit a peak LHCP gain of approximately $\:6.2\:\text{d}\text{B}\text{i}\text{c}$ at $\:28\:\text{G}\text{H}\text{z}$, indicating a strong directional response with efficient circular polarization. The gain remains above $\:5\:\text{d}\text{B}\text{i}\text{c}$ across a broad portion of the $\:27\--29\:\text{G}\text{H}\text{z}$ band, demonstrating robust performance within the intended operational range. The smooth variation of the gain response, without abrupt fluctuations, confirms the stability and integrity of the circularly polarized radiation even under experimental conditions. Additionally, the alignment of the gain peak with the center of the impedance-matching band ensures optimal radiation efficiency and polarization purity at the desired frequency.

These results confirm that the antenna, when configured for LHCP operation, not only achieves the intended polarization reconfigurability but also maintains a high and stable gain across the circularly polarized band. This makes it suitable for advanced millimeter-wave communication systems requiring polarization diversity, such as satellite communication and adaptive wireless networks.

**Fig. 19:**
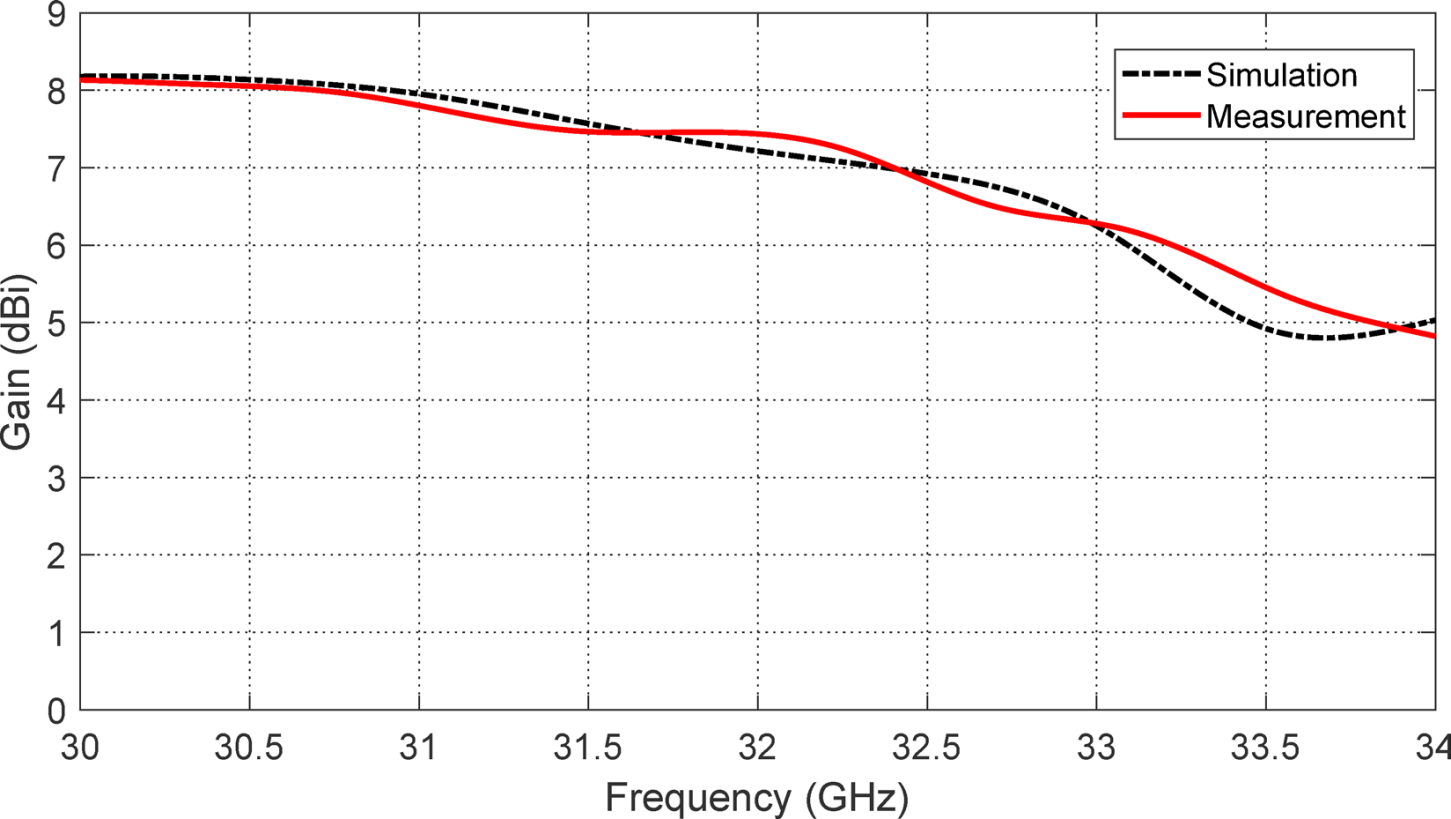
Comparison between the simulation and experimental results for the frequency response of the gain of the proposed reconfigurable antenna over the frequency range $\:30-34\:\text{G}\text{H}\text{z}$ (the range around the upper frequency band of operation) when the PIN diodes $\:{D}_{A1}$ and $\:{D}_{B1}$ are activated (forward biased) whereas the PIN diodes $\:{D}_{A2}$ and $\:{D}_{B2}$ are deactivated. The gain is $\:7.2\:\text{d}\text{B}\text{i}$ at $\:32.3\:\text{G}\text{H}\text{z}$.

Figure 19 presents a comparison between the simulated and experimental gain responses of the proposed reconfigurable antenna over the frequency range 30–34 GHz, focusing on the upper frequency band of operation. In this configuration, the PIN diodes $\:{D}_{A1}$ and $\:{D}_{B1}$ are activated (forward biased), while $\:{D}_{A2}$ and $\:{D}_{B2}$ are deactivated (reverse biased), enabling the antenna to operate in a linearly polarized mode suitable for high-frequency applications. Both the simulation and measurement curves exhibit a clear peak in gain performance centered around $\:32.3\:\text{G}\text{H}\text{z}$. At this frequency, the antenna achieves a maximum gain of approximately $\:7.2\:\text{d}\text{B}\text{i}$, indicating a highly directional radiation pattern with effective power concentration. The agreement between the simulated and measured gain curves confirms the accuracy of the design and the success of the fabrication and testing processes. The gain remains above 6.5 dBi across a substantial portion of the $\:32\--32.6\:\text{G}\text{H}\text{z}$ band, which aligns well with the impedance matching and linear polarization characteristics observed in previous figures. This consistent high-gain behavior across the operational band indicates that the antenna maintains stable and efficient performance within the designated upper band. Additionally, the smooth variation in the gain curve and the close alignment between simulation and measurement validate the robustness of the antenna’s electromagnetic behavior and confirm that the reconfigurable structure functions effectively at higher frequencies. The slight discrepancies between simulation and measurement are within acceptable limits and are likely due to fabrication tolerances, measurement uncertainties, or minor losses in the experimental setup.

Overall, Fig. 19 demonstrates that the proposed antenna, in its reconfigured state, successfully achieves high-gain linearly polarized radiation in the $\:32\--32.6\:\text{G}\text{H}\text{z}$ band, making it a strong candidate for high-frequency applications in millimeter-wave communication systems such as 5G, satellite links, and radar systems.

#### Experimental results for the axial ratio

**Fig. 20:**
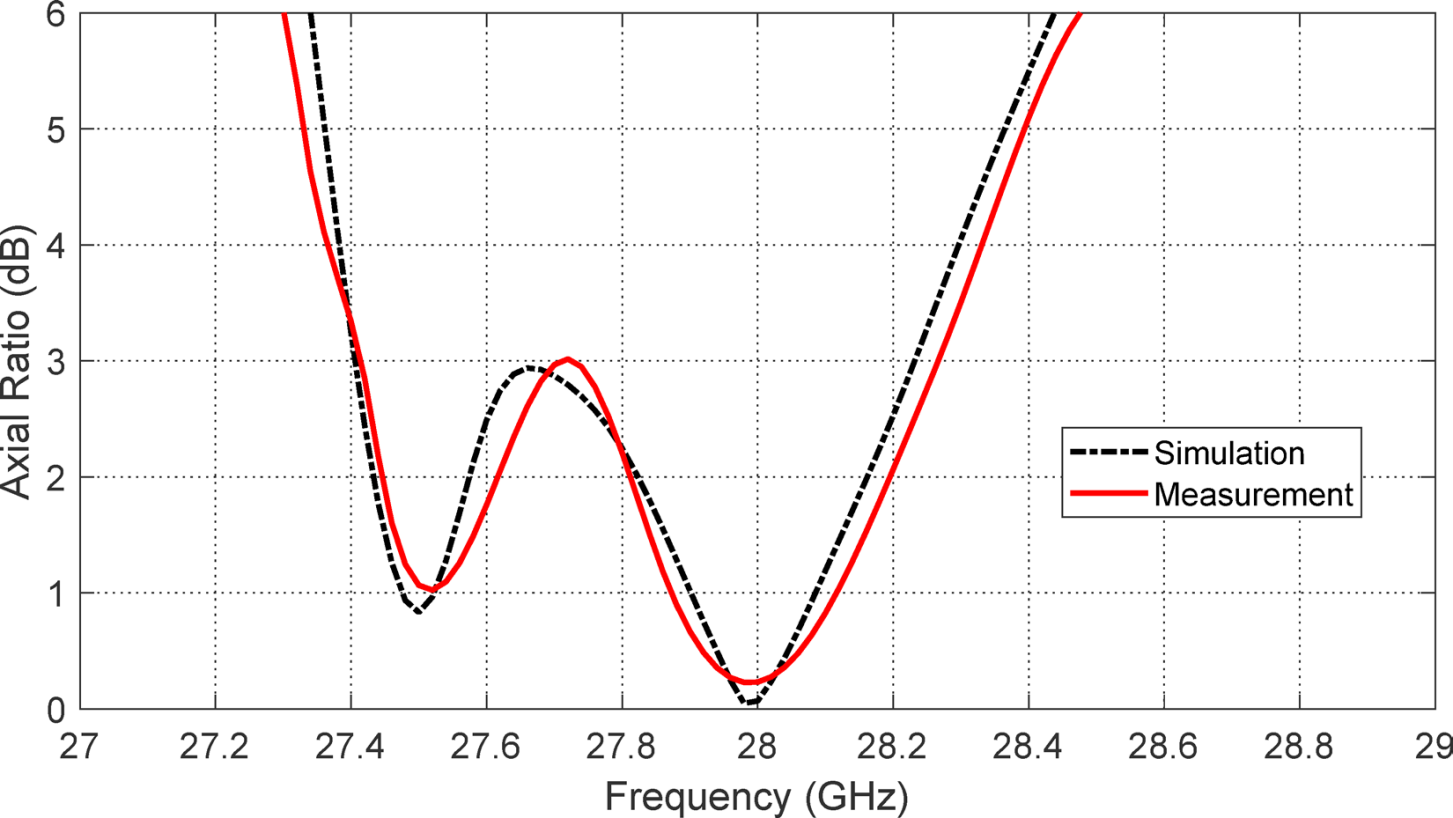
Comparison between the simulation and experimental results for the frequency response of the axial raito of the proposed reconfigurable antenna when the PIN dioes $\:{D}_{A1}$ and $\:{D}_{B1}$ are activated (forward biased) whereas the PIN diodes $\:{D}_{A2}$ and $\:{D}_{B2}$ are deactivated.

Figure 20 presents a comparison between the simulated and measured frequency responses of the axial ratio for the proposed reconfigurable antenna when operating in the LHCP mode. This mode is achieved by forward-biasing PIN diodes $\:{D}_{A1}$ and $\:{D}_{B1}$ while reverse-biasing diodes $\:{D}_{A2}$ and $\:{D}_{B2}$. The axial ratio is a critical metric that quantifies the degree of circular polarization, with values below 3 dB indicating effective circular polarization. Both the simulated and measured curves clearly demonstrate that the antenna achieves circular polarization over a substantial frequency range. Specifically, the 3 dB axial ratio bandwidth extends from $\:27.42\:\text{G}\text{H}\text{z}$ to $\:28.22\:\text{G}\text{H}\text{z}$, resulting in an $\:800\:\text{M}\text{H}\text{z}$-wide frequency band suitable for LHCP operation. This wide CP bandwidth highlights the effectiveness of the antenna geometry, feed structure, and integrated reconfigurable elements in supporting high-fidelity circular polarization over the desired frequency range. The simulation and experimental results show good agreement in both the center frequency and the overall bandwidth of the CP response, with only slight deviations attributable to fabrication tolerances, material property variations, and measurement uncertainties. The minimum axial ratio is close to $\:0.2\:\text{d}\text{B}$ at $\:28\:\text{G}\text{H}\text{z}$, confirming high polarization purity and excellent circular polarization performance. The measured axial ratio remains well below the 3 dB threshold across most of the CP band, validating the antenna’s practical capability to deliver circularly polarized radiation in real-world conditions. This characteristic is particularly beneficial for mm-wave communication systems and satellite links, where polarization mismatch can lead to significant signal degradation.

In summary, Fig. 20 confirms that the proposed reconfigurable antenna achieves a robust and wide 800-MHz circular polarization bandwidth from $\:27.42\:\text{G}\text{H}\text{z}$ to $\:28.22\:\text{G}\text{H}\text{z}$ in LHCP mode, with excellent agreement between simulation and measurement, making it well-suited for high-performance, polarization-agile mm-wave applications.

## Simulation results

This section presents the numerical results obtained from full-wave electromagnetic simulations conducted using CST Studio Suite. The analysis focuses on evaluating the antenna’s performance under different polarization states. Key parameters such as surface current distribution, circularly polarized radiation patterns, and radiation efficiency are examined to confirm the effectiveness of the proposed reconfiguration technique. The detailed results are provided in the following subsections.

### Comparison with baseline antenna performance

**Fig. 21:**
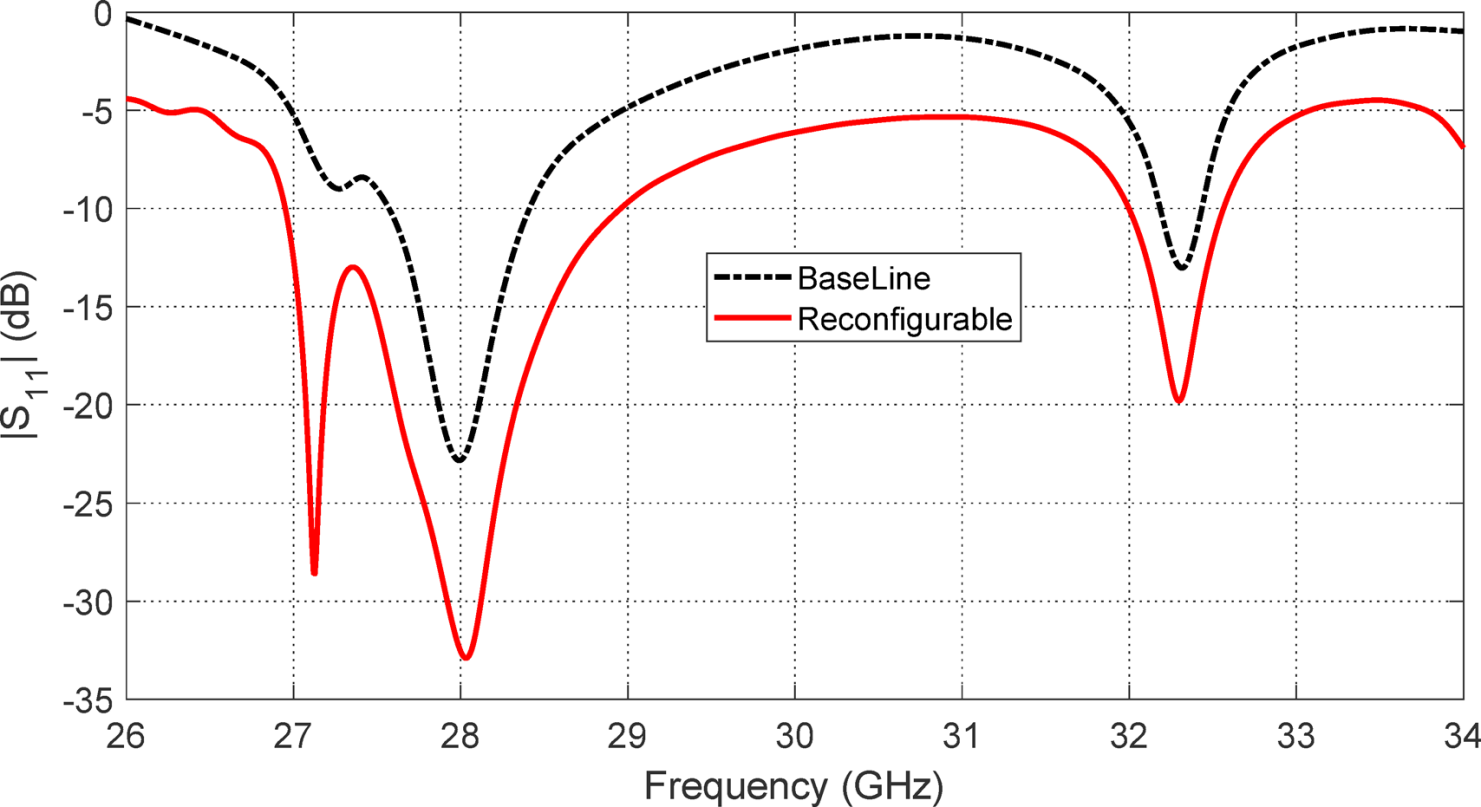
Comparison between the frequency responses of $\:\left|{S}_{11}\right|$ of the baseline antenna and the reconfigurable antenna ($\:{D}_{A1}$ and $\:{D}_{B1}$​ are forward biased while $\:{D}_{A2}$ and $\:{D}_{B2}$​ are reverse biased).

**Fig. 22:**
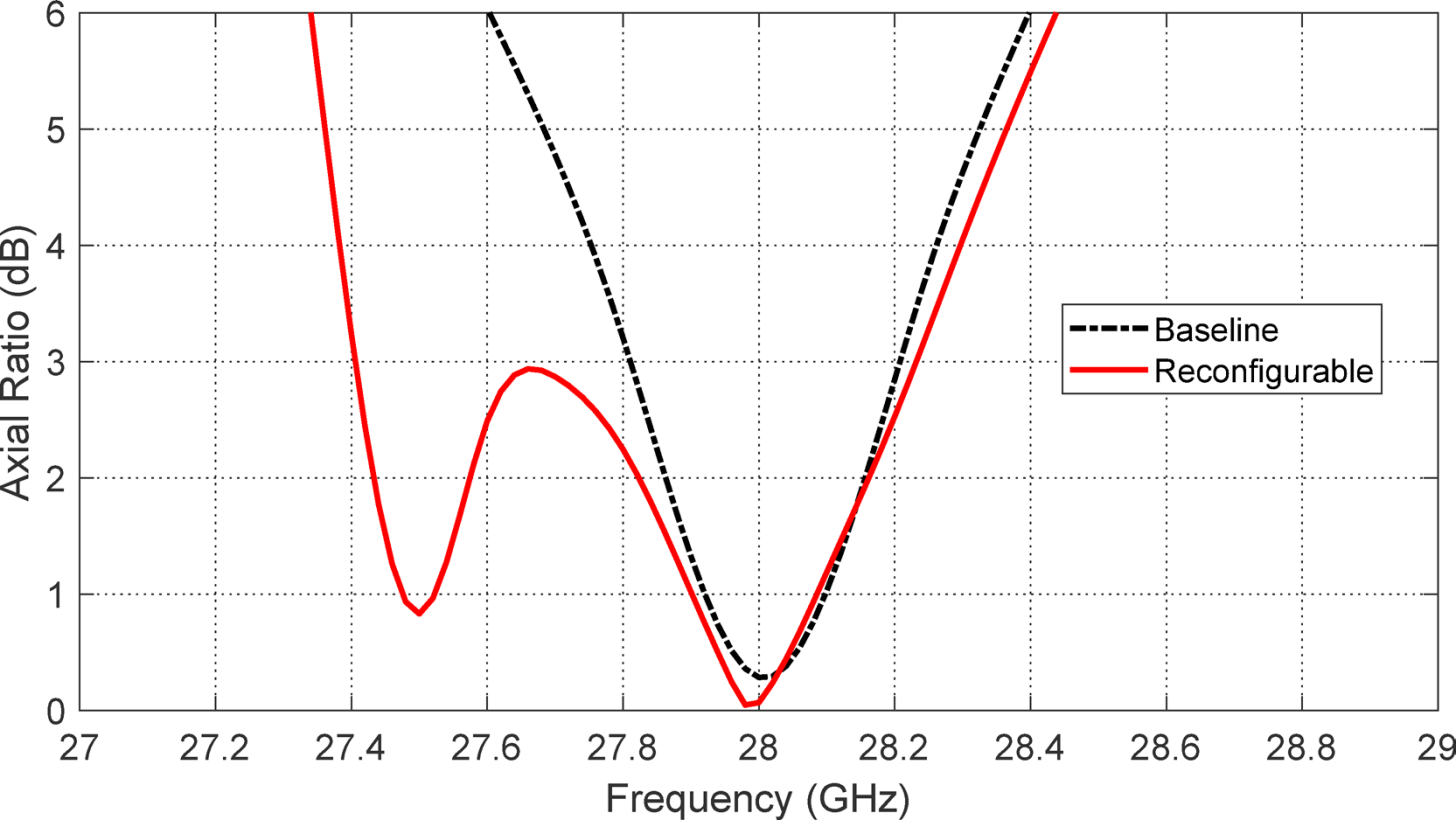
Comparison between the frequency responses of the axial ratio of the baseline antenna and the reconfigurable antenna ($\:{D}_{A1}$ and $\:{D}_{B1}$​ are forward biased while $\:{D}_{A2}$ and $\:{D}_{B2}$​ are reverse biased).

The design process of the proposed antenna began with a baseline configuration, where the radiating patch and feed network were optimized without the inclusion of PIN diodes. When the diodes were subsequently introduced, frequency shifts were observed in both the resonant frequencies and the location of the axial-ratio (AR) minimum. To mitigate these effects, the antenna geometry was further refined to restore the baseline resonance and polarization performance. Interestingly, the refined design with integrated diodes not only recovered the original behavior but also provided noticeable performance enhancements. In particular, impedance matching bandwidth is significantly broadened compared to the baseline antenna as shown in Fig. 21. Also, the 3-dB AR bandwidth is significantly improved as shown in Fig. 22. These results highlight that the integration of PIN diodes, along with careful design refinement, does not degrade the antenna’s inherent performance but instead contributes to enhanced bandwidth characteristics while enabling polarization reconfigurability.

### Surface current distribution

**Fig. 23:**
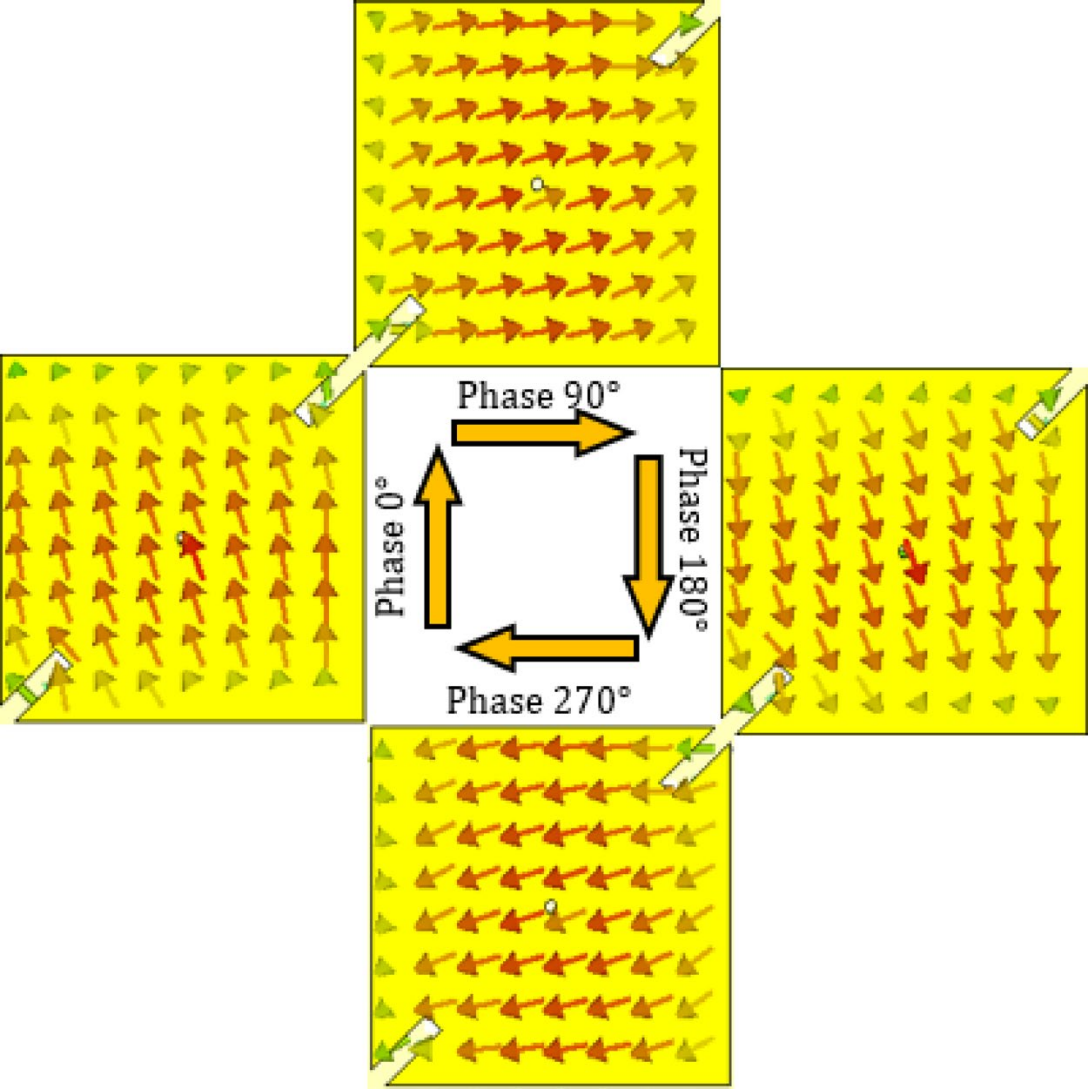
Surface current distributions at $\:28\:\text{G}\text{H}\text{z}$ on the patch at different time phases ($\:0^\circ\:$, $\:90^\circ\:$, $\:180^\circ\:$, and $\:270^\circ\:$) when the PIN diodes $\:{D}_{A1}$ and $\:{D}_{B1}$ are activated and $\:{D}_{A2}$ and $\:{D}_{B2}$ are deactivated, corresponding to the LHCP radiation mode of the proposed antenna.

**Fig. 24:**
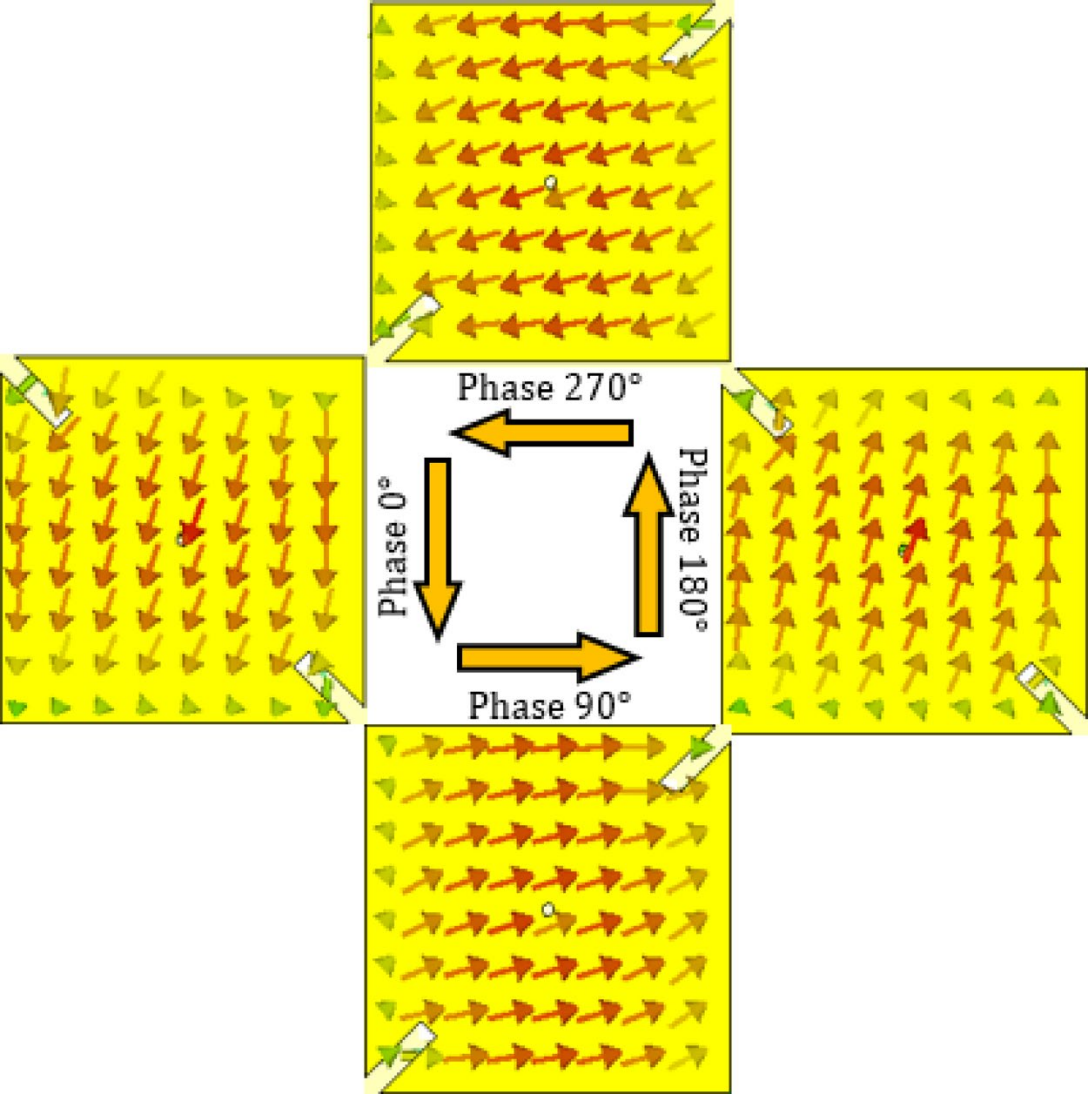
Surface current distributions at $\:28\:\text{G}\text{H}\text{z}$ on the patch at different time phases ($\:0^\circ\:$, $\:90^\circ\:$, $\:180^\circ\:$, and $\:270^\circ\:$) when the PIN diodes $\:{D}_{A2}$ and $\:{D}_{B2}$ are activated and $\:{D}_{A1}$ and $\:{D}_{B1}$ are deactivated, corresponding to the RHCP radiation mode of the proposed antenna.

Figures 23 and 24 illustrate the reconfigurable circular polarization capability of the proposed antenna at 28 GHz by showing the time-evolving surface current distributions on the patch for the two diode biasing states. These distributions provide clear physical insight into how the antenna achieves polarization switching between left-hand circular polarization (LHCP) and right-hand circular polarization (RHCP).

In Fig. 23, diodes $\:{D}_{A1}$ and $\:{D}_{B1}$​ are forward biased (“ON”), while $\:{D}_{A2}$ and $\:{D}_{B2}$​ are reverse biased (“OFF”), activating Branch 1 of the feed. The resulting surface current distribution on the patch evolves with time phases 0, 90, 180, and 270°, showing a clockwise rotation. This clockwise rotation corresponds to the excitation of two orthogonal modes ($\:{TM}_{10}$​ and $\:{TM}_{01}$​) with a 90° phase difference, producing a radiated field that is left-hand circularly polarized.

In contrast, Fig. 24 shows the case when diodes $\:{D}_{A2}$ and $\:{D}_{B2}$​ are forward biased and $\:{D}_{A1}$ and $\:{D}_{B1}$​ are turned off, thereby activating Branch 2 of the feed. The surface current vectors again exhibit a 90° progressive phase shift across the patch edges, but this time the current rotates counter-clockwise as the time phase advances. This counter-clockwise behavior indicates that the orthogonal resonant modes are excited with the opposite phase relationship, thus generating right-hand circular polarization.

The observed current rotations in Figs. 23 and 24 validate the dual-branch feed design: by selectively biasing the integrated PIN diodes, the antenna determines which patch edge is excited first, thereby controlling the direction of current rotation and consequently the polarization sense. This mechanism ensures that the required conditions for circular polarization, two orthogonal modes of equal magnitude and 90° time quadrature, are consistently satisfied in both polarization states.

Compared with conventional CP generation methods that rely on hybrid couplers, sequential feeding, or mechanical rotation, the proposed approach achieves electronic polarization reconfiguration in a compact structure without additional circuitry. Thus, Figs. 23 and 24 not only confirm the LHCP/RHCP switching capability but also highlight the efficiency of the diode-controlled feed network in realizing dynamic polarization control at millimeter-wave frequencies.

### Radiation patterns of the circularly polarized field components

**Fig. 25:**
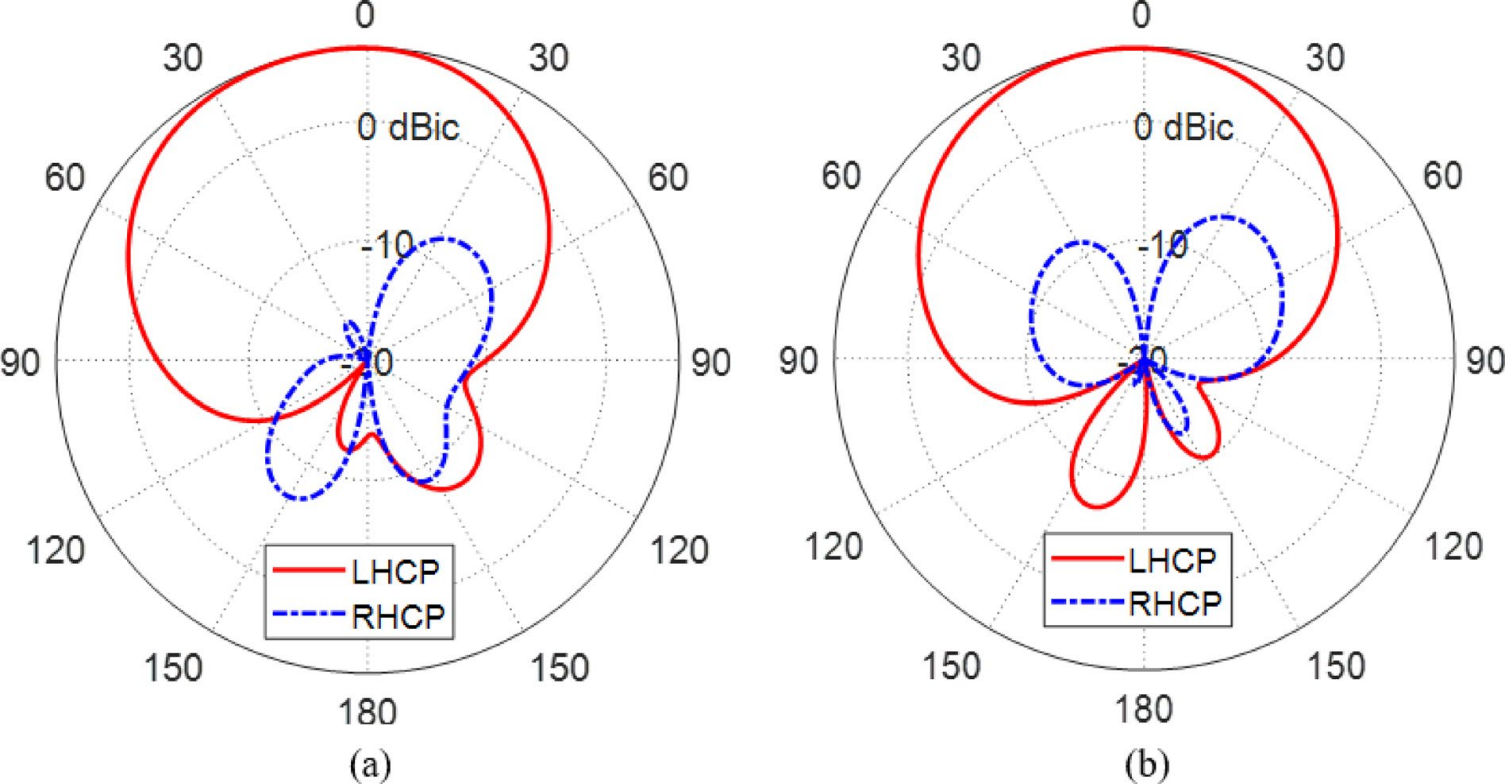
Gain patterns of the co-polarized (LHCP) and cross-polarized (RHCP) field components at $\:28\:\text{G}\text{H}\text{z}$ obtained by simulation for the proposed reconfigurable antenna when the PIN diodes $\:{D}_{A1}$ and $\:{D}_{B1}$ are activated whereas the PIN diodes $\:{D}_{A2}$ and $\:{D}_{B2}$ are deactivated. (**a**) Plane $\:\varphi\:=0^\circ\:$ (**b**) Plane $\:\varphi\:=90^\circ\:$.

**Fig. 26:**
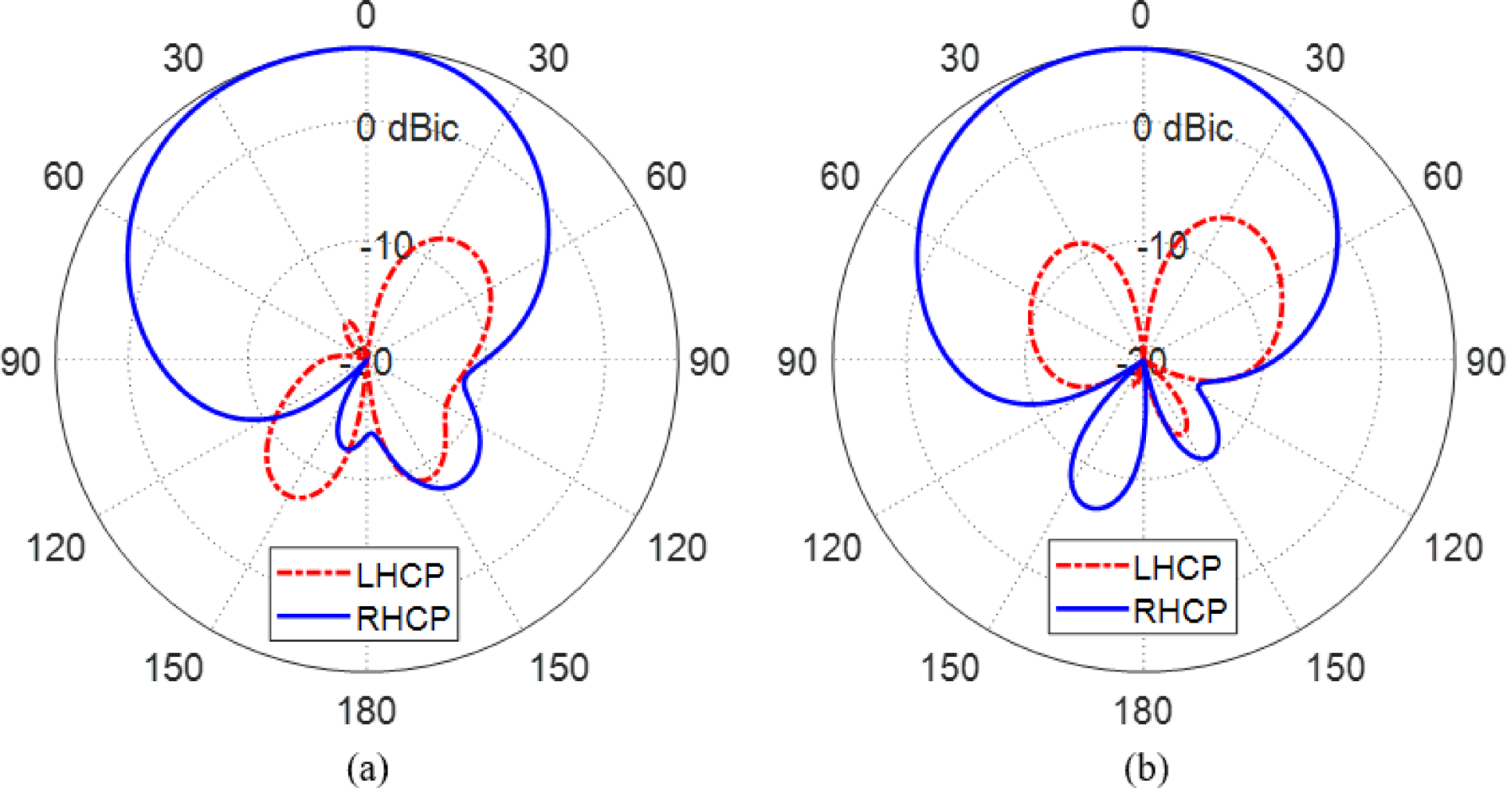
Gain patterns of the co-polarized (RHCP) and cross-polarized (LHCP) field components at $\:28\:\text{G}\text{H}\text{z}$ obtained by simulation for the proposed reconfigurable antenna when the PIN diodes $\:{D}_{A1}$ and $\:{D}_{B1}$ are deactivated whereas the PIN diodes $\:{D}_{A2}$ and $\:{D}_{B2}$ are activated. (**a**) Plane $\:\varphi\:=0^\circ\:$ (**b**) Plane $\:\varphi\:=90^\circ\:$.

Figures 25 and 26 together highlight the reconfigurability of the proposed antenna in terms of polarization control at $\:28\:\text{G}\text{H}\text{z}$, demonstrating its ability to switch between left-hand circular polarization (LHCP) and right-hand circular polarization (RHCP) by selectively activating different sets of PIN diodes.

In Fig. 25, the antenna operates in LHCP mode when the PIN diodes $\:{D}_{A1}$ and $\:{D}_{B1}$ are activated (forward biased), and $\:{D}_{A2}$ and $\:{D}_{B2}$ are deactivated (reverse biased). The gain patterns in both $\:\varphi\:=0^\circ\:$ and $\:\varphi\:=90^\circ\:$ planes show a dominant co-polarized LHCP component with a peak gain directed toward the boresight ($\:\theta\:=0^\circ\:$), while the cross-polarized RHCP component remains significantly suppressed throughout the angular range. The high cross-polarization discrimination affirms that the antenna radiates efficiently in LHCP with minimal interference from the orthogonal polarization, thus validating its intended operation in this state.

Conversely, Fig. 26 presents the antenna’s performance when reconfigured to RHCP mode by deactivating $\:{D}_{A1}$ and $\:{D}_{B1}$ and activating $\:{D}_{A2}$ and $\:{D}_{B2}$. The resulting gain patterns at $\:\varphi\:=0^\circ\:$ and $\:\varphi\:=90^\circ\:$ show a strong RHCP co-polarized component, again centered around the boresight direction, while the LHCP cross-polarized field is suppressed with a cross-polarization level well below the main lobe. The symmetry and consistency of the patterns confirm the robustness of the antenna’s reconfiguration mechanism and its capability to maintain desirable radiation characteristics under both polarization states.

Together, Figs. 25 and 26 clearly demonstrate the antenna’s effective reconfigurability between LHCP and RHCP modes using simple electronic control of the PIN diodes. This capability is essential for adaptive or bidirectional satellite and wireless communication systems where polarization agility enhances link reliability and reduces signal degradation due to mismatched polarization. The maintained directional gain and high polarization purity in both configurations confirm the design’s success in achieving reliable dual-sense circular polarization through reconfiguration.

### Antenna efficiency

**Fig. 27:**
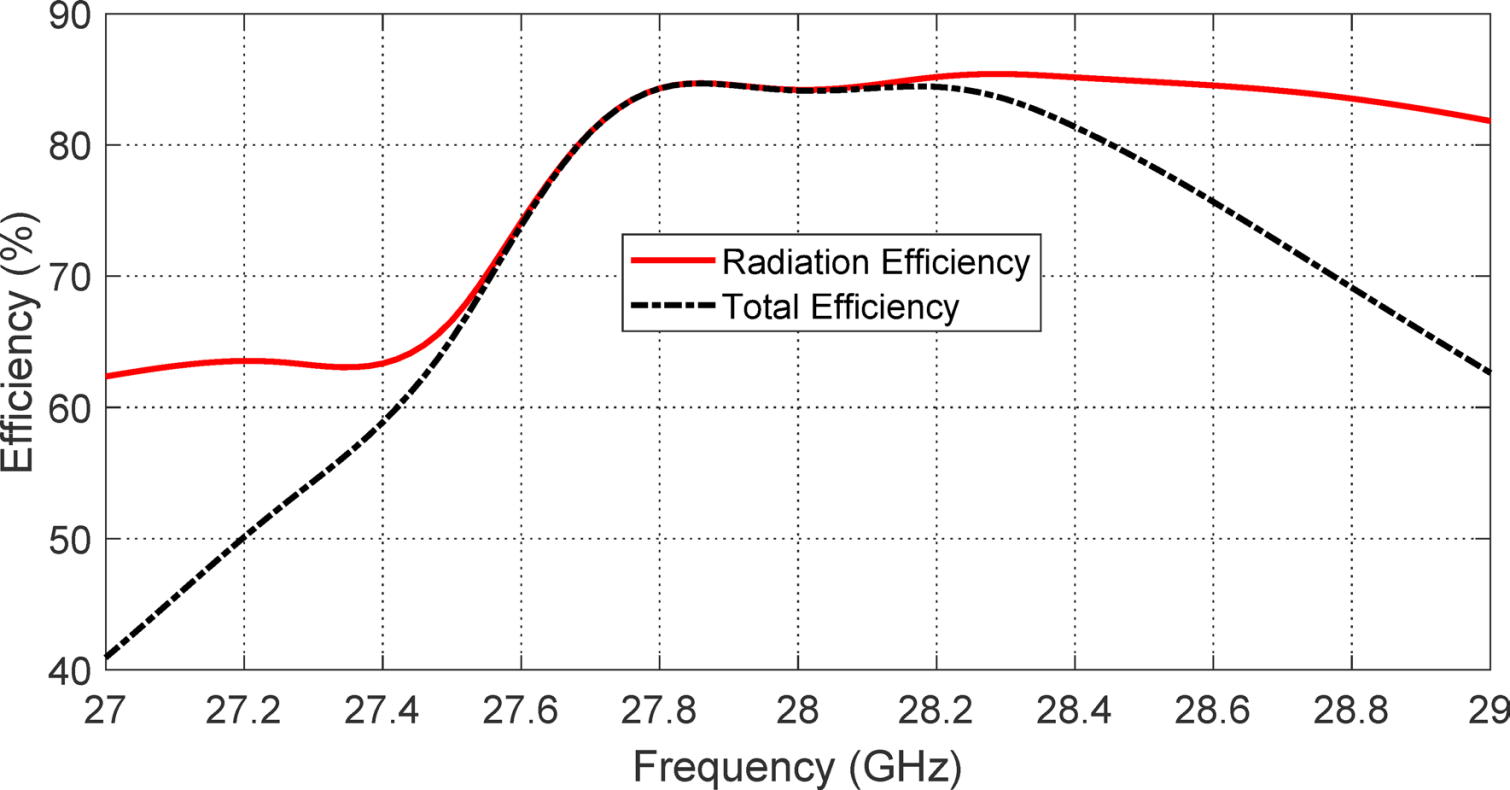
Variation of the total and radiation efficiencies of the proposed reconfigurable antenna over the frequency range 27–29 GHz as obtained by simulation antenna when the PIN diodes $\:{D}_{A1}$ and $\:{D}_{B1}$ are activated whereas the PIN diodes $\:{D}_{A2}$ and $\:{D}_{B2}$ are deactivated.

**Fig. 28:**
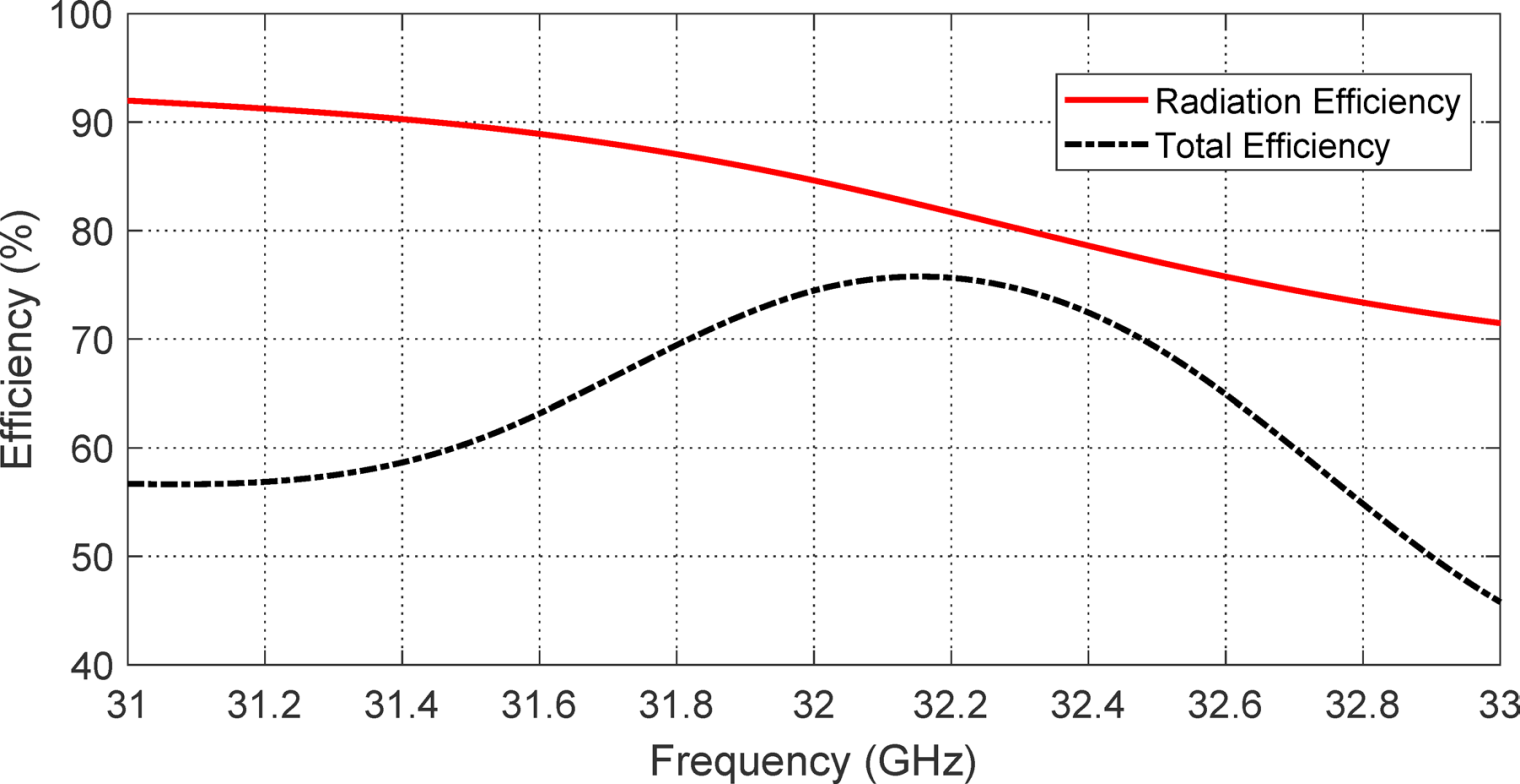
Variation of the total and radiation efficiencies of the proposed reconfigurable antenna over the frequency range 31–33 GHz as obtained by simulation antenna when the PIN diodes $\:{D}_{A1}$ and $\:{D}_{B1}$ are activated whereas the PIN diodes $\:{D}_{A2}$ and $\:{D}_{B2}$ are deactivated.

Figures 27 and 28 collectively illustrate the efficiency performance of the proposed reconfigurable antenna in the two distinct polarization modes enabled through PIN diode switching. These figures present the simulated variations of total and radiation efficiencies across the respective operating frequency bands corresponding to LHCP and RHCP radiation.

Figure 27 focuses on the frequency range of $\:27\--29\:\text{G}\text{H}\text{z}$, where the antenna is configured for LHCP radiation by activating PIN diodes $\:{D}_{A1}$ and $\:{D}_{B1}$ while deactivating $\:{D}_{A2}$ and $\:{D}_{B2}$. Within this lower band, the antenna demonstrates stable and closely matched total and radiation efficiencies. At 28 GHz, both efficiencies reach a peak value of approximately $\:84\%$, indicating that the majority of the input power is effectively converted into radiated energy with minimal Ohmic or dielectric losses. This high and consistent efficiency across the band highlights the effectiveness of the antenna design in maintaining performance under the LHCP configuration.

Figure 28 shows the corresponding efficiency variation over the upper operating band of $\:31\--33\:\text{G}\text{H}\text{z}$ when the diode configuration is switched to activate $\:{D}_{A2}$ and $\:{D}_{B2}$ (for RHCP radiation) and deactivate $\:{D}_{A1}$ and $\:{D}_{B1}$. In this case, the radiation efficiency at $\:32.3\:\text{G}\text{H}\text{z}$ remains relatively high at about $\:82\%$, but the total efficiency drops slightly to $\:76\%$. This drop reflects a modest increase in loss, likely attributable to the increased series resistance and parasitics introduced by the active components or slightly less optimal current distribution in this configuration.

Together, Figs. 27 and 28 confirm that the antenna maintains good efficiency performance in both reconfigurable states, supporting reliable operation in either LHCP or RHCP mode depending on the PIN diode biasing. The results also validate the practicality of the proposed design for dual-band, polarization-reconfigurable applications while maintaining relatively high efficiency in each mode.

## Comparison with similar designs

To contextualize the proposed antenna, we compare its performance with six recently reported reconfigurable antennas designed for mm-wave communication. Reference^14^ presents a compact loop antenna that enables both polarization and bandwidth reconfigurability using PIN diodes, achieving an axial ratio below 3 dB over the 25–29 GHz range and a gain of up to 9 dBi. Reference^25^ describes a dual-band reconfigurable MIMO antenna operating at 28 and 38 GHz, where PIN diodes are used to control polarization states in each band, delivering good isolation and axial ratio performance below 3 dB across both bands. Reference^26^ proposes a 10 mm × 10 mm square microstrip antenna with symmetrical biasing for polarization switching using PIN diodes, demonstrating stable axial ratio performance and 7.5 dBi gain. In^27^, a polarization-reconfigurable antenna array designed for 5G millimeter-wave applications is introduced, featuring PIN-diode-based switching and a wide operational range from 27 to 30 GHz with a peak gain of 8.4 dBi. Reference^28^ achieves high-quality circular polarization with a single PIN-diode switching mechanism and demonstrates a low axial ratio of 1.7 dB at 29 GHz. Reference^29^ employs MEMS switches to implement polarization reconfigurability over a wideband 58–62 GHz range, with an axial ratio below 3.5 dB and compact geometry suitable for integration in high-frequency systems.

The comparison in Table 2 clearly demonstrates the superior performance of the proposed antenna in several key aspects. Notably, it offers one of the smallest form factors (12 × 12 × 0.25 mm³), which is advantageous for compact millimeter-wave (mm-wave) front-end integration, especially in 5G and 6G transceiver modules. Unlike most prior designs, which focus on polarization reconfiguration only, the proposed antenna supports lower-band polarization reconfiguration, offering extended functionality across both 27–29 GHz and 32–32.3 GHz, thereby enhancing frequency agility.

In terms of radiation characteristics, the proposed design achieves a low axial ratio (< 3 dB) over a wide frequency span and an exceptional value of 0.2 dB at 28 GHz, which indicates excellent circular polarization purity—outperforming all other listed works. Additionally, it maintains high gain levels (6.2 dBic at 28 GHz, 7.2 dBi at 32.3 GHz) and a notable radiation efficiency of 88%, placing it among the most efficient designs.

Furthermore, the proposed antenna achieves this performance using PIN diodes, which offer fast switching and compatibility with standard fabrication processes, while avoiding more complex or costly control components such as MEMS switches.

Overall, the proposed antenna offers a well-balanced combination of compactness, dual-band operation, high gain, excellent polarization purity, and efficiency, making it highly suitable for next-generation mm-wave communication systems.

**Table 2:** Performance comparison of recent polarization-reconfigurable Mm-wave antennas.

Property	^14^	^25^	^26^	^27^	^28^	^29^	Proposed
Dimensions $\:\text{m}\text{m}\times\:\text{m}\text{m}\times\:\text{m}\text{m}$	$\:8\times\:6\times\:1$	$\:15\times\:12\times\:0.8$	$\:10\times\:10\times\:1.6$	$\:15\times\:15\times\:0.79$	$\:10.2\times\:14.1\times\:0.78$	$\:5.4\times\:5.2\times\:0.4$	$\:12\times\:12\times\:0.25$
Relative dimensions $\:\frac{x}{\lambda\:}\times\:\frac{y}{\lambda\:}\times\:\frac{z}{\lambda\:}$	$\:0.64\times\:0.48\times\:0.08$	$\:1.4\times\:1.12\times\:0.075$	$\:0.88\times\:0.88\times\:0.14$	$\:1.35\times\:1.35\times\:0.07$	$\:0.99\times\:1.36\times\:0.075$	$\:1.04\times\:1.0\times\:0.077$	$\:1.08\times\:1.08\times\:0.025$
Frequency band(s) (GHz)	24–30 GHz	28 38	26.5–29.5	27–30	29	58–62	27–29 32–32.3
Reconfiguration type	Polarization + Bandwidth	Dual-band Polarization	Polarization	Polarization	Polarization	Polarization	Lower-band Polarization
Control element	PIN diodes	PIN diodes	PIN diodes	PIN diodes	PIN diodes	MEMS switches	PIN diodes
Axial ratio	< 3 dB (25–29 GHz)	< 3 dB (Both bands)	< 3.5 dB	< 3 dB	1.7 dB at 29 GHz	< 3.5 dB	< 3 dB 27.4–8.2 GHz 0.2 dB at 28 GHz
Gain	7–9 dBi	6.2–8.1 dBi	7.5 dBi	8.4 dBi	6.5 dBi	5.9 dBi	6.2 dBic at 28 GHz 7.2 dBi at 32.3 GHz
Rad. efficiency	80%	70%	85%	85%	90%	82%	88%
Applied design Method	Slot-loaded patch	Multilayer patch	Slotted rectangular patch	AMC-backed design	Compact slot antenna	MEMS-based multilayer	Multilayer PCB integration
Design complexity	Medium	High	Medium	High	Medium	High	Medium

As summarized in Table 2, the proposed antenna compares favorably with recent polarization-reconfigurable mm-wave antennas in terms of gain, radiation efficiency, and compactness. By including the applied design method and design complexity in the comparison, it becomes clear that while some prior works^25,27,29^ achieve good performance, they often rely on multilayer or MEMS-based structures that significantly increase fabrication difficulty and biasing complexity. In contrast, the proposed antenna employs a straightforward multilayer PCB integration method that achieves high efficiency (88%) and competitive gain (6.2–7.2 dBi) while maintaining only a medium level of design complexity. This balance between simplicity and performance highlights the practicality of the proposed approach for real-world mm-wave communication systems, where both ease of fabrication and reliable reconfigurability are essential.

## Conclusion

This paper presents a compact, polarization-reconfigurable, dual-band antenna suitable for millimeter-wave communication systems. The antenna is implemented as a square microstrip patch notched at two opposite corners, with four PIN diodes integrated into the notches to control the surface current distribution and enable polarization reconfiguration. The antenna has a DGS) to enhance the circular polarization and increase the bandwidth. Depending on the diode biasing state, the antenna produces either LHCP or RHCP in the lower band (27–29 GHz), while maintaining linear polarization in the upper band (32–32.6 GHz).The antenna exhibits good impedance matching across both bands, with $\:|\text{S}₁₁|\:<\:\--10\:\text{d}\text{B}$, and a minimum axial ratio of 0.2 dB at $\:28\:\text{G}\text{H}\text{z}$, demonstrating excellent circular polarization purity. Simulated gain values exceed $\:7\:\text{d}\text{B}\text{i}$ in both configurations. At 28 GHz, both the radiation and total efficiencies reach $\:84\%$, while at $\:32.3\:\text{G}\text{H}\text{z}$, the efficiencies are $\:82\%$ and $\:76\%$, respectively. The surface current distribution and gain patterns confirm the polarization behavior under different diode states. The experimental measurements show good agreement with the results obtained by simulation. These results validate the proposed notched square patch antenna as a viable, efficient, and adaptable solution for future reconfigurable millimeter-wave systems. As a future extension of the present work, it is proposed to construct MIMO antenna systems based on the developed single-element antenna in order to enhance the overall communication system performance for future generations of mm-wave applications.

## Data Availability

The datasets used and/or analyzed during the current study available from the corresponding author on reasonable request.
